# The METTL5-TRMT112 *N*^6^-methyladenosine methyltransferase complex regulates mRNA translation *via* 18S rRNA methylation

**DOI:** 10.1016/j.jbc.2022.101590

**Published:** 2022-01-14

**Authors:** Caraline Sepich-Poore, Zhong Zheng, Emily Schmitt, Kailong Wen, Zijie Scott Zhang, Xiao-Long Cui, Qing Dai, Allen C. Zhu, Linda Zhang, Arantxa Sanchez Castillo, Haiyan Tan, Junmin Peng, Xiaoxi Zhuang, Chuan He, Sigrid Nachtergaele

**Affiliations:** 1 Department of Chemistry, University of Chicago, Chicago, Illinois, USA; 2 Institute for Biophysical Dynamics, University of Chicago, Chicago, Illinois, USA; 3 Department of Biochemistry and Molecular Biology, University of Chicago, Chicago, Illinois, USA; 4 University of Chicago Medical Scientist Training Program, Chicago, Illinois, USA; 5 Department of Neurobiology, University of Chicago, Chicago, Illinois, USA; 6 Center for Proteomics and Metabolomics, St Jude Children's Research Hospital, Memphis, Tennessee, USA; 7 Departments of Structural Biology and Developmental Neurobiology, St Jude Children's Research Hospital, Memphis, Tennessee, USA; 8 Howard Hughes Medical Institute, University of Chicago, Chicago, Illinois, USA

**Keywords:** ribosomal RNA, RNA methylation, m^6^A methylation, translation regulation, methyltransferase, posttranscriptional regulation, 2′OMe, 2′O-methylation, CLIP, cross-linking-assisted immunoprecipitation, IP, immunoprecipitation, KO, knockout, m1A, N1-methyladenosine, m6A, N6-methyladenosine, m6,6A, N6,6-methyladenosine, m7G, N7-methylguanosine, qPCR, quantitative PCR, rRNA, ribosomal RNA, TE, translation efficiency

## Abstract

Ribosomal RNAs (rRNAs) have long been known to carry chemical modifications, including 2′*O*-methylation, pseudouridylation, *N*^6^-methyladenosine (m^6^A), and *N*^6,6-^dimethyladenosine. While the functions of many of these modifications are unclear, some are highly conserved and occur in regions of the ribosome critical for mRNA decoding. Both 28S rRNA and 18S rRNA carry single m^6^A sites, and while the methyltransferase ZCCHC4 has been identified as the enzyme responsible for the 28S rRNA m^6^A modification, the methyltransferase responsible for the 18S rRNA m^6^A modification has remained unclear. Here, we show that the METTL5-TRMT112 methyltransferase complex installs the m^6^A modification at position 1832 of human 18S rRNA. Our work supports findings that TRMT112 is required for METTL5 stability and reveals that human METTL5 mutations associated with microcephaly and intellectual disability disrupt this interaction. We show that loss of METTL5 in human cancer cell lines and in mice regulates gene expression at the translational level; additionally, *Mettl5* knockout mice display reduced body size and evidence of metabolic defects. While recent work has focused heavily on m^6^A modifications in mRNA and their roles in mRNA processing and translation, we demonstrate here that deorphanizing putative methyltransferase enzymes can reveal previously unappreciated regulatory roles for m^6^A in noncoding RNAs.

Chemical modifications on RNA are a critical facet of gene expression regulation. Historically, modifications on tRNA and rRNA have been thought to have high stoichiometry and be relatively static, while work in the last decade on mRNA modifications suggests they are often substoichiometric and more dynamic (1). rRNA is heavily modified with numerous chemical marks, including pseudouridine, 2′*O*-methylation (2′*O*Me), *N*^7^-methylguanosine (m^7^G), *N*^1^-methyladenosine (m^1^A), *N*^6^-methyladenosine (m^6^A), and *N*^6,6^-methyladenosine (m^6,6^A) (2, 3, 4). While these modifications are thought to play critical structural roles and many of the regulatory enzymes have been identified, it is often difficult to assign specific functions to individual modifications due to the numerous interactions between the three rRNAs and more than 80 protein components that form the ribosome. While some modifications play structural roles in ribosome assembly, others may regulate translation of specific transcripts. Disruption of rRNA modification processes has been implicated in a class of developmental disorders called ribosomopathies (5, 6, 7, 8). Interestingly, though the ribosome is ubiquitously essential for translating protein, ribosomopathies often manifest as tissue-specific disorders, the molecular mechanisms of which we do not understand in many cases (9).

The METTL protein family is a class of S-adenosyl-methionine-dependent methyltransferases, with over 30 family members that methylate DNA (10), RNA (11, 12, 13, 14), and protein (15, 16) substrates. Some, such as METTL3, METTL14, and METTL16, have well-characterized functions as RNA m^6^A methyltransferases (11, 12), but others remain poorly understood. Notably, mutations in many of these enzymes, including those whose functions are unknown, have been implicated in human diseases such as developmental abnormalities and cancers (17, 18, 19). Revealing METTL protein substrate specificity, activity, and function is a critical first step toward understanding how mutations in these enzymes cause human disease. More specifically, mutations in METTL5 have been implicated in developmental abnormalities including microcephaly, intellectual disabilities, and attention deficit hyperactivity disorder (ADHD), but until recently very little was known about METTL5 function (18, 20). We were intrigued by this connection when METTL5 appeared in proteomics experiments aimed at identifying novel proteins with methyltransferase activity.

While this work was in progress, multiple studies revealed that a complex containing METTL5 and TRMT112 m^6^A-methylates 18S rRNA in flies, mice, and humans (21, 22, 23, 24). METTL-5 in *Caenorhabditis elegans* also carries out this function, but not in the context of a complex with TRMT112, as *C. elegans* lack a TRMT112 homologue (25, 26). Interestingly, the role of METTL5 in protein translation differs across the different model organisms studied. Consistent with these reports, we found that METTL5 forms a complex with TRMT112 to m^6^A methylate 18S rRNA in human cell lines and in mice. We further found that TRMT112 is critical for stabilizing METTL5 at the protein level, and that depletion of TRMT112 is sufficient to reduce METTL5 protein levels. Catalytically inactive METTL5 mutants can retain this critical association with TRMT112, but METTL5 mutations derived from human patients dramatically reduce this interaction. While we did not see global changes in protein translation upon METTL5 depletion, we found evidence of dysregulated translation of specific transcripts. To complement our cellular studies, we generated *Mettl5* knockout mice (*Mettl5*^*−/−*^) and validated the loss of 18S rRNA m^6^A1832 in tissues from these mice. Consistent with Ignatova *et al.* and Wang *et al.*, we observed smaller body size in our *Mettl5*^*−/−*^ mouse model (22, 27). We did not observe the significant behavioral changes that have been previously described, but RNA sequencing of mouse tissues revealed evidence of lipid metabolic defects that have not yet been reported. Ribosome profiling of mouse livers suggested that the effects of Mettl5 loss predominantly occur at the level of translation. Altogether, we propose that METTL5 may regulate the translation of transcripts associated with lipid metabolism, resulting in metabolic dysregulation that may play a role in the developmental phenotypes seen in human patients.

## Results

### METTL5 is an m^6^A methyltransferase stabilized by TRMT112

**Figure 1:**
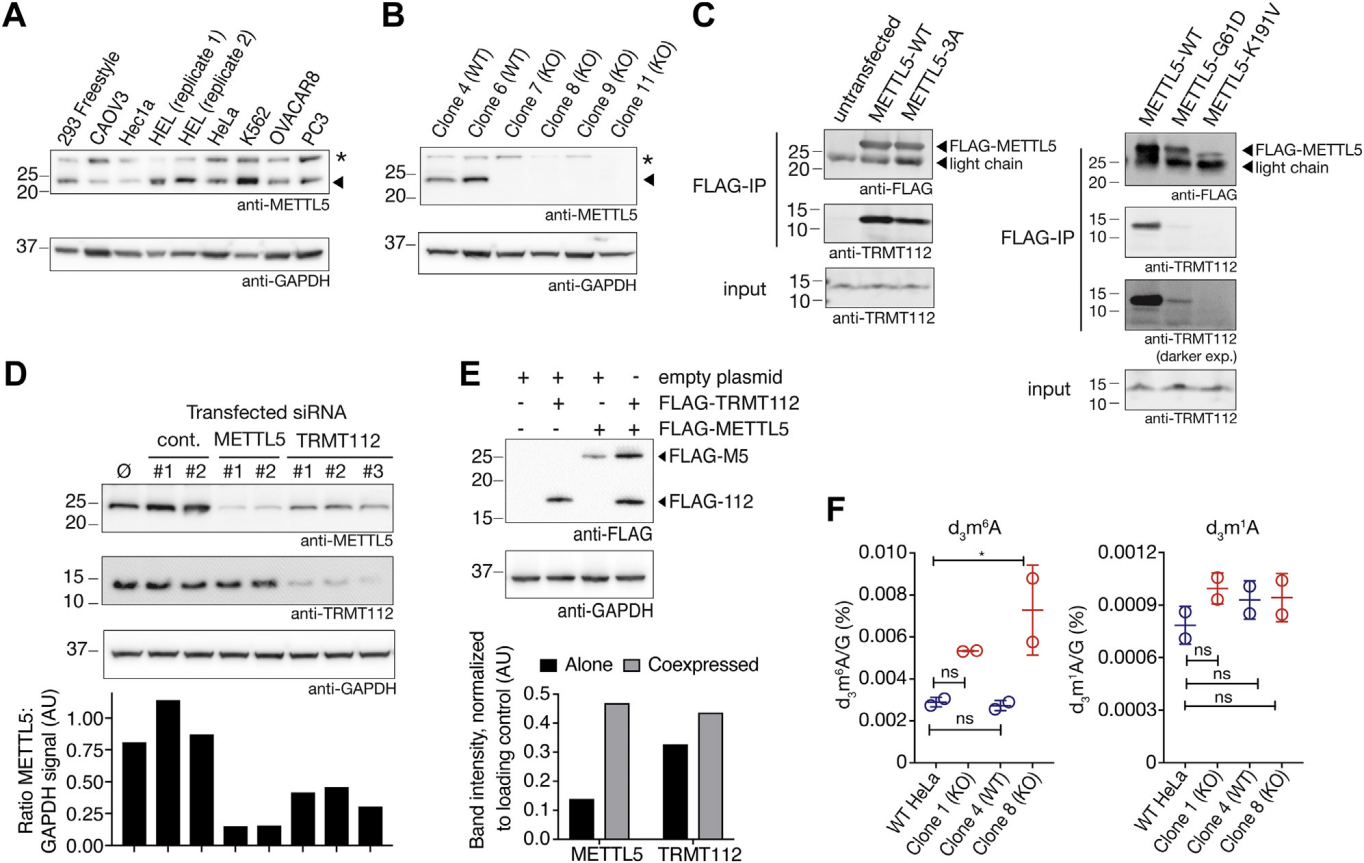
**METTL5 is an m**^**6**^**A methyltransferase stabilized by TRMT112.***A*, METTL5 expression in cell lines as evaluated by Western blot (*top*) with anti-GAPDH loading control (*bottom*). HEL (replicate 1) and (replicate 2) represent two different passages of HEL cells. *Arrowhead* signifies METTL5 band; ∗ signifies background band. *B*, Western blot analysis of METTL5 levels in wild-type (WT) and knockout (KO) HeLa samples, each expanded from a single isolated clone (*top*) with anti-GAPDH loading control (*bottom*). *Arrowhead* signifies METTL5 band; ∗ signifies background band. *C*, coimmunoprecipitations of TRMT112 with FLAG-METTL5. *Left*, anti-FLAG (*top*) and anti-TRMT112 (*middle*) Western blots in anti-FLAG immunoprecipitation samples. Input TRMT112 levels shown on *bottom*. Lanes from *left* to *right* are untransfected HeLa, HeLa transfected with FLAG-METTL5, and HeLa transfected with the FLAG-METTL5-3A mutant. *Right*, anti-FLAG (*top*) and anti-TRMT112 (*middle*) Western blots in anti-FLAG immunoprecipitation samples. Input TRMT112 levels shown on *bottom*. Labels: METTL5-WT, METTL5-3A, METTL5-G61D, METTL5-K191V: HeLa transfected with FLAG-METTL5-WT, FLAG-METTL5-3A, FLAG-METTL5-G61D, and FLAG-METTL5-K191Vfs∗10, respectively. *D*, *top*, Western blots for METTL5, TRMT112, and GAPDH (loading control) levels upon siRNA knockdown for METTL5 and TRMT112. Labels: Ø, untransfected HeLa cells; cont., HeLa transfected with two different nontargeting control siRNAs; METTL5, HeLa transfected with two different siRNAs targeting METTL5; TRMT112, HeLa transfected with three different siRNAs targeting TRMT112. *Bottom*, ratio of METTL5 to GAPDH signal intensity as quantified by Fiji software. *E*, *top*, Western blot analysis of FLAG-METTL5 and FLAG-TRMT112 expression from HeLa transfected with labeled combinations of vectors, with GAPDH loading control. *Bottom*, band intensity normalized to GAPDH band intensity, as quantified by Fiji software. *F*, LC-MS/MS analysis of d_3_m^6^A (*left*) and d_3_m^1^A (*right*) levels normalized to guanosine from *in vitro* methyltransferase reactions performed on total RNA isolated from METTL5-WT and METTL5-KO HeLa clones. n = 2 replicate reactions, mean and s.e.m. plotted, analyzed by one-way ANOVA, comparing all samples to HeLa WT with Dunnett’s test for multiple comparisons. ns: not significant, ∗*p* <0.05. All indicated band sizes in Western blots are in kilodaltons (kDa).

METTL5 is a member of the METTL family of S-adenosyl-methionine-dependent methyltransferases, which is not found in yeast but is conserved in higher eukaryotes ranging from *C. elegans*, to mice, to humans. While the activities and functions of some METTL proteins have been elucidated over the last decade (11, 12, 13, 14), many remain poorly understood. RNA methyltransferases, particularly the METTL3-METTL14 complex, have been demonstrated to have numerous cellular functions through their methyltransferase activity, which raises the question as to how less well-characterized METTL proteins might regulate cellular processes. METTL5 drew our attention during a biochemical screen that was aimed at identifying novel RNA methyltransferase activity through biochemical fractionation followed by a liquid chromatography coupled to tandem mass spectrometry (LC-MS/MS)-based assay (see Supplementary Note, Fig. S6, Table S8 for more details). We surveyed commonly used cancer cell culture lines by Western blot and found that HeLa, HEL, and K562 cells showed relatively higher METTL5 expression (Fig. 1*A*). HeLa cells are the only adherent line of these three and are easily transfected, making biochemical and imaging studies straightforward. Thus, we generated METTL5 knockout (KO) HeLa lines using CRISPR-Cas9 and two sets of two guide RNAs to generate deletions across two different exons (Fig. S1*A*). After puromycin selection, single clones were isolated, expanded, and tested for METTL5 expression by Western blot (Figs. 1*B* and S1*A*). Through this process, we isolated both KO lines and clones in which METTL5 expression was unaffected (*e.g.*, clones 4 and 6), which serve as controls throughout this work.

Detailed studies of other m^6^A methyltransferases have revealed specific motifs required for the catalytic activity of these enzymes, including the ^395^DPPW^398^ motif in the m^6^A mRNA methyltransferase METTL3 (28). METTL5 contains a chemically similar ^127^NPPF^130^ motif, which led us to hypothesize that METTL5 may also be an m^6^A methyltransferase. However, through our efforts to use *in vitro* methylation assays with tagged, overexpressed METTL5 protein to validate its methyltransferase activity, we noted lower-than-typical yields of purified METTL5 protein from both bacterial and mammalian expression systems and low modification fractions in our *in vitro* assays (data not shown). Since methyltransferases such as METTL3-METTL14 function as a complex, this prompted us to perform proteomic analysis of METTL5-binding proteins using FLAG-METTL5. For these experiments, we turned to FreeStyle 293-F cells, which produce more robust overexpression of METTL5 than the HeLa cells we used previously. TRMT112 was a particularly intriguing candidate binding protein (Fig. S1*B* and Table S1), because it is known to bind and regulate other methyltransferases such as AlkBH8 (a tRNA methyltransferase) (29) and HemK2 (a protein methyltransferase) (30). Indeed, subsequent experiments confirmed a direct interaction between METTL5 and TRMT112 (Fig. 1*C*, left) (21).

Mutation of a portion of the METTL5 putative catalytic motif ^127^NPP^129^ to AAA (amino acids 127–129, abbreviated METTL5-3A), predicted to abolish its m^6^A methyltransferase activity, weakened but did not completely disrupt this interaction. Since mutations in METTL5 have also been found in human patients with intellectual disability and microcephaly (18, 20) we introduced two of these human variants into our FLAG-METTL5 construct, G61D and K191Vfs∗10 (a 2 bp deletion that results in a K > V point mutation and premature stop). In transient transfection overexpression experiments, FLAG-METTL5-G61D expressed at levels approximately 50 to 80% the level of expression of FLAG-METTL5-WT. Despite substantial expression of METTL5-G61D, the interaction with TRMT112 was compromised and the level of TRMT112 bound to FLAG-METTL5-G61D was approximately 5 to 15% relative to that bound to FLAG-METTL5-WT (Fig. 1*C*, right). This reduced interaction could potentially be explained by structural changes introduced by disrupting flexibility of a loop region (Fig. S1*C*). FLAG-METTL5-K191Vfs∗10 expressed at much lower levels, suggesting that this truncated protein may not fold properly (Fig. 1*C*, right; METTL5-K191V). Based on a previous report, the third variant identified in human patients, METTL5-R115Nfs∗19, expresses only at very low levels, so we did not test its expression or interaction with TRMT112 (20).

The interaction with TRMT112 stabilizes METTL5 protein, as siRNA knockdown of TRMT112 reduced expression of METTL5 to approximately half relative to negative controls (Fig. 1*D*), though less than direct siRNA knockdown of METTL5. The stabilization effect occurs at the protein level, as METTL5 transcript levels were unaffected by TRMT112 knockdown (Fig. S1*D*). Conversely, coexpression of FLAG-METTL5 with FLAG-TRMT112 in HeLa cells significantly increased FLAG-METTL5 protein expression compared with solely expressing FLAG-METTL5 (Fig. 1*E*). Coexpression of FLAG-TRMT112 with FLAG-METTL5 also substantially increased protein expression yields from FreeStyle 293-F cells, allowing us to purify FLAG-METTL5-FLAG-TRMT112 complex for *in vitro* methyltransferase assays. Using deuterated S-adenosyl methionine (d_3_SAM), we performed *in vitro* methyltransferase assays with copurified FLAG-METTL5-FLAG-TRMT112, using total RNA isolated from METTL5-WT and METTL5-KO HeLa cells. Analysis of deuterated, methylated nucleosides by LC-MS/MS revealed d_3_m^6^A in all four samples (Fig. 1*F*). RNA from METTL5-KO cells accumulated approximately double the amount of d_3_m^6^A as RNA isolated from METTL5-WT cells, consistent with the idea that total RNA from METTL5-KO cells has less m^6^A to begin with. Parallel measurements in these same samples showed tenfold lower levels of d_3_m^1^A that remained consistent across all samples. Taken together, these results suggest that METTL5 is an m^6^A RNA methyltransferase that requires TRMT112 for stability, as corroborated by van Tran *et al.* (21).

### 18S rRNA is a major substrate of the METTL5-TRMT112 complex

**Figure 2:**
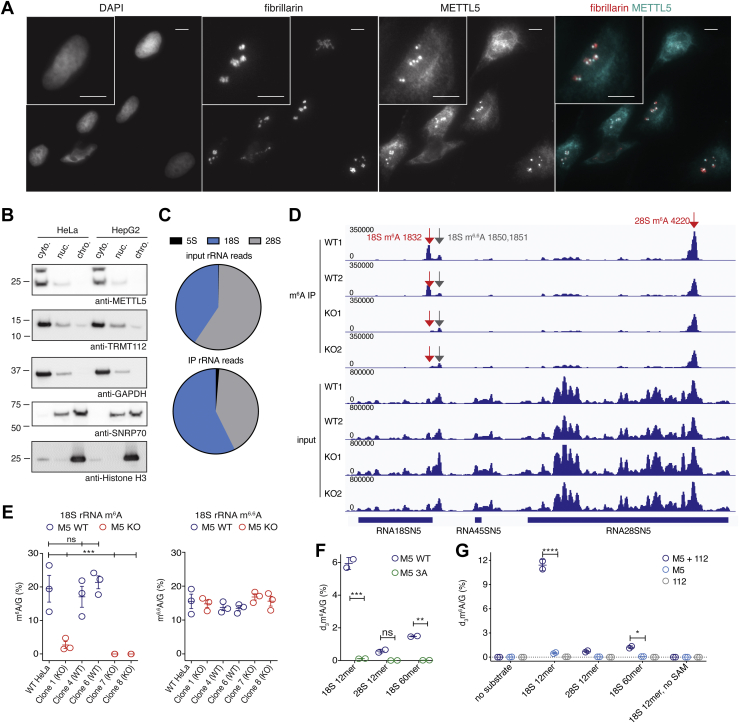
**METTL5 is predominantly cytoplasmic and methylates 18S rRNA.***A*, fluorescence microscopy images of HeLa cells stained with DAPI (nuclear stain), anti-fibrillarin antibody (nucleolar marker), and anti-METTL5 antibody. *Right panel* shows merged image. Scale bars: 10 μm (panels and inset). *B*, localization of METTL5 and TRMT112 in chromatin-associated, nuclear, and cytoplasmic cellular fractions in HeLa and HepG2 by Western blot. Loading and fractionation controls: GAPDH (cytoplasmic), SNRP70 (nuclear), Histone H3 (chromatin-associated). *C*, pie charts of reads aligned to 5S, 18S, or 28S rDNA from HeLa input and METTL5 CLIP samples. *D*, visualization of reads aligned to the pre-45S locus from input and anti-m^6^A immunoprecipitated Me-RIP-seq samples from HeLa METTL5-WT and METTL5-KO, adapted from Integrated Genomics Viewer. *E*, levels of m^6^A (*left*) and m^6,6^A (*right*), normalized to G, obtained by LC-MS/MS of 40-nt probe-purified segments of 18S rRNA surrounding the m^6^A 1832 site from HeLa METTL5-WT and METTL5-KO. n = 3 reactions per condition, mean and s.e.m. plotted. Comparisons of all samples to the WT HeLa control were tested by one-way ANOVA with Dunnett’s test for multiple comparisons. *F*, LC-MS/MS analysis of d_3_m^6^A levels normalized to G from *in vitro* methyltransferase reactions performed on 18S and 28S-mimicking probes with TRMT112 and either wild-type METTL5 (M5 WT) or catalytic mutant METTL5-3A (M5 3A). *G*, LC-MS/MS analysis of d_3_m^6^A levels normalized to G from *in vitro* methyltransferase reactions performed on 18S and 28S probes with METTL5 (M5) alone, TRMT112 alone (112), or METTL5-TRMT112 complex (M5+112). *F* and *G*, were analyzed using two-way ANOVA to compare M5 WT with M5 3A (with Sidak test for multiple comparisons in (*F*)) and METTL5 with METTL5-TRMT112 (with Tukey test for multiple comparisons in (*G*)). n = 2 reactions per condition, mean and s.e.m. ∗*p* < 0.05, ∗∗*p* < 0.01, ∗∗∗*p* < 0.005. ∗∗∗∗*p* < 0.0001. (all other comparisons not statistically significant and labeled [ns], or not shown). All indicated band sizes in Western blots are in kilodaltons (kDa).

To begin to understand the function of the METTL5-TRMT112 complex in cells, we first sought to identify its subcellular distribution. Fluorescence microscopy using antibodies targeting endogenous METTL5 revealed nuclear puncta that colocalized with the nucleolar protein fibrillarin, but also had a more diffuse staining pattern in the cytoplasm (Fig. 2*A*). This distribution was also verified using biochemical fractionation, which revealed that, while there was no detectable METTL5 associated with chromatin, there is a small pool of nuclear METTL5, with the majority of the protein localized to the cytoplasm (Fig. 2*B*). This subcellular localization is particularly intriguing given the large difference observed in d_3_m^6^A methylation of total RNA isolated from WT and KO cells (Fig. 1*F*), suggesting a relatively large pool of RNA to be methylated by METTL5-TRMT112. The nucleolus is a critical hub for the processing and assembly of ribosome components, and rRNAs are known to be m^6^A methylated. Both 28S and 18S rRNAs have an m^6^A site (3), and while ZCCHC4 has been identified as the methyltransferase for 28S rRNA A4220 (31, 32), the methyltransferase for 18S rRNA was unknown when we initiated these studies.

To identify RNA substrates of METTL5, we performed cross-linking-assisted immunoprecipitation (CLIP) of FLAG-tagged METTL5 followed by high-throughput sequencing of bound RNAs. To maximize the likelihood of success, we incorporated 4-thiouridine to facilitate cross-linking (33) (see Experimental procedures for a detailed protocol). High-throughput sequencing revealed a slight enrichment for 18S rRNA transcripts in the immunoprecipitate (IP) relative to input (Fig. 2*C*), as well as other enriched transcripts (Fig. S2*A* and Table S2). m^6^A-seq in METTL5-WT and METTL5-KO HeLa cells showed loss of an m^6^A peak near adenosine 1832 in 18S rRNA while the adjacent m^6,6^A1850 and m^6,6^A1851 sites remained unchanged (Fig. 2*D* and Table S3), supporting the hypothesis that 18S rRNA is a major substrate of the METTL5-TRMT112 complex. We then applied biotinylated DNA probes complementary to the region of 18S rRNA containing both m^6^A1832 and the neighboring m^6,6^A1850,1851 sites to capture and purify these fragments from METTL5-WT and METTL5-KO cells for LC-MS/MS analysis (31). Results confirmed that METTL5-TRMT112 methylates 18S rRNA A1832, as this fragment showed dramatically lower levels of m^6^A in all KO lines tested relative to WT (Fig. 2*E*, left panel). In contrast, the nearby m^6,6^A sites showed no significant change among these cell lines (Fig. 2*E*, right panel), demonstrating that 18S rRNA processing is not dramatically altered and that these modifications are regulated independently.

To verify that METTL5-TRMT112 is not only necessary, but also sufficient for deposition of 18S rRNA m^6^A1832, we expressed and purified FLAG-METTL5-WT and catalytically inactive FLAG-METTL5-3A with FLAG-TRMT112 from FreeStyle 293-F cells and tested their activity on RNA probes containing 18S or 28S rRNA sequences, using LC-MS/MS to measure d_3_m^6^A levels. Neither FLAG-METTL5-WT nor FLAG-METTL5-3A complexes could efficiently methylate a 28S rRNA 12-mer (Fig. 2*F*), suggesting specificity for 18S rRNA sequence. Moreover, only METTL5-WT-TRMT112 complexes could effectively methylate 18S rRNA 12-mer or 60-mer probes, while the METTL5-3A mutant showed nearly undetectable activity. Using this 18S rRNA 12mer substrate, we then performed a more detailed assessment of the individual contributions of METTL5 and TRMT112 in methyltransferase activity. While copurified FLAG-METTL5 and FLAG-TRMT112 could effectively methylate 18S rRNA probes, this activity was undetectable when either protein was purified individually or when d_3_SAM was left out of the reaction (Fig. 2*G*). The loss of METTL5 activity in the absence of TRMT112 is likely the result of poorly folded METTL5 protein, as suggested by increased stability of METTL5 in the presence of TRMT112 (Fig. 1, *D* and *E*). Taken together, our results are consistent with recent reports (21, 22, 24) that the METTL5-TRMT112 complex m^6^A methylates 18S rRNA and that the ^127^NPP^129^ motif is critical for its catalytic activity.

To assess whether METTL5-TRMT112 may have other RNA substrates, we delved more deeply into the other transcripts enriched in our METTL5 CLIP experiment (Fig. S2, *A* and *B* and Table S2), which included both coding and noncoding RNAs. Cross-referencing these METTL5-bound RNAs with differentially methylated transcripts from our m^6^A-seq experiment (Fig. S2*C* and Table S3) revealed only 25 overlapping transcripts (Fig. S2*D*). While the most prominent motif in the m^6^A peaks overall was GGACU, suggesting that most m^6^A peaks were METTL3/METTL14 dependent (11), the 25 transcripts in common between the two datasets showed enrichment for UAA, the motif containing the m^6^A site in 18S rRNA. Thus, although it has been reported that 18S rRNA is the only METTL5-TRMT112 substrate (21), it remains possible that other targets may exist. Notably, the coding transcripts identified in our CLIP experiment are enriched for genes involved in mitochondrial biogenesis and function (Fig. S2*B*). RNA-seq analysis of differentially expressed transcripts in METTL5-WT and METTL5-KO HeLa cells also revealed enrichment for small-molecule transport and lipid and cholesterol biosynthesis pathways (Fig. S2*E* and Table S4). These preliminary connections to metabolism and lipid biosynthesis, both liver-based functions, led us to generate HepG2 METTL5-KO cell lines, which may reflect gene expression pathways in the liver more closely (Fig. S2, *F* and *G*).

### METTL5 regulates translation of a subset of transcripts

**Figure 3:**
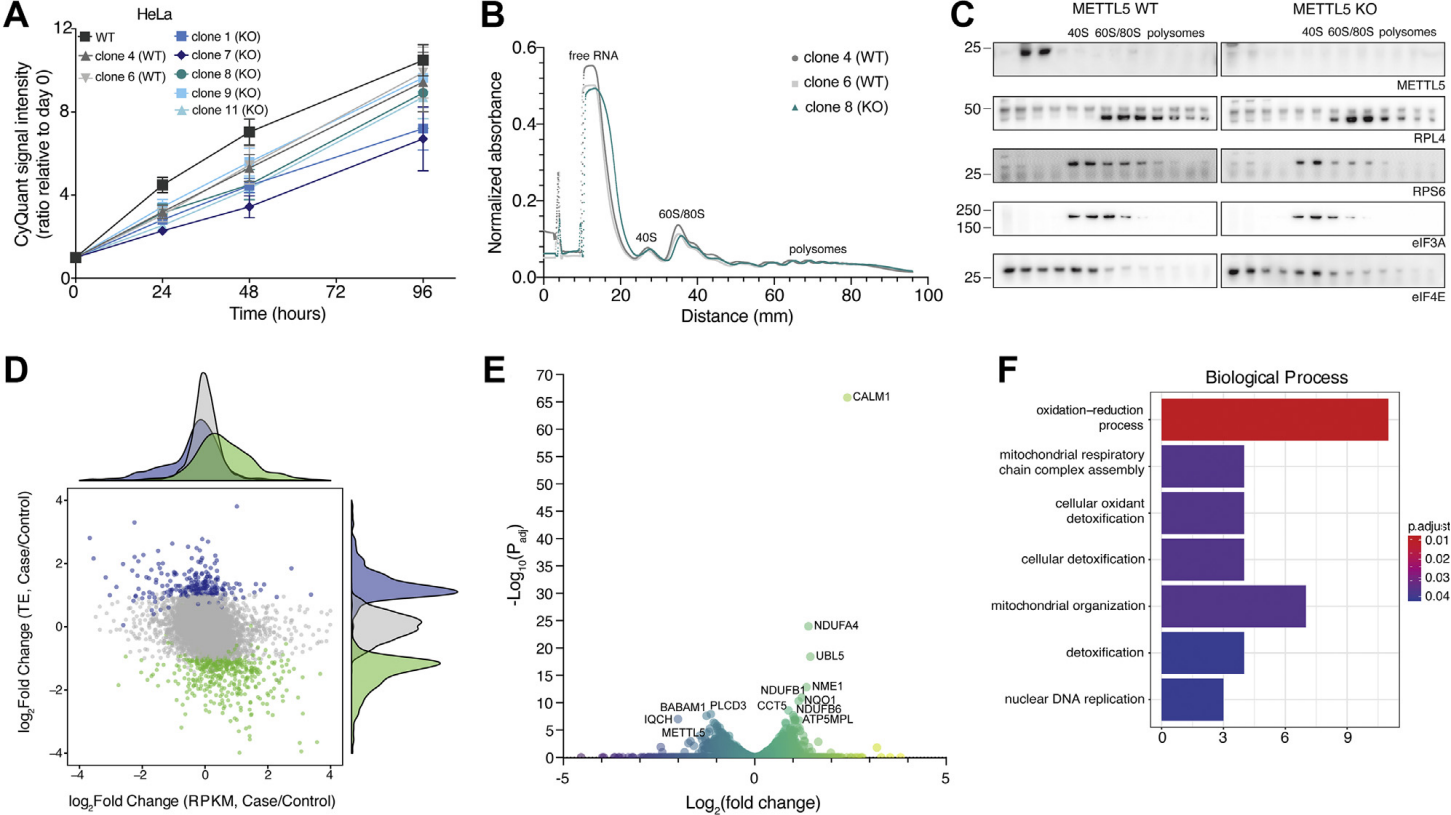
**METTL5 affects cell proliferation and metabolism through differential translation of specific transcripts.***A*, cell proliferation of HeLa METTL5-WT and METTL5-KO cells over time as measured by CyQuant assay. Means (*squares*) and SDs (*bars*) are indicated for four replicate wells per condition. *B*, polysome profiles from HeLa METTL5-WT and METTL5-KO cells as measured by normalized absorbance over a 5 to 50% sucrose gradient. *C*, Western blots of polysome fractions from panel *B* for METTL5, RPL4 (large ribosomal subunit marker), RPS6 (small ribosomal subunit marker), and eIF3A and eIF4E (translation initiation factors). *D*, effects of METTL5 knockout on translation and transcription as displayed by log_2_(fold change) of translation efficiency (TE) *versus* log_2_(fold change) of reads per kilobase of transcript per million mapped reads (RPKM) from ribosome profiling input and ribosome-protected fragment sequencing of HepG2 cells. Case = METTL5-KO, Control = METTL5-WT. *E*, volcano plot of the negative log_10_ of adjusted *p*-value *versus* log_2_(fold change) of normalized gene expression from ribosome profiling of HepG2 METTL5-KO *versus* METTL5-WT cells. *F*, biological process gene ontology (GO) functional enrichment analysis for translationally upregulated transcripts in HepG2 METTL5-KO compared with METTL5-WT cells. *D* and *F* are modified from output of RiboToolKit (61). All indicated band sizes in Western blots are in kilodaltons (kDa).

Through the course of our experiments with both HeLa and HepG2 cells, we noted that METTL5-KO cells tended to grow more slowly than the corresponding METTL5-WT cells. To measure this difference directly, cell growth curves were monitored over the course of 96 h using DNA-dye-based CyQuant cell proliferation assays. In both HeLa and HepG2 cells, METTL5-KO cell lines grew more slowly than WT cells, with a small but consistent difference in growth rate (Figs. 3*A* and S3*A*). rRNAs are heavily modified with numerous chemical groups that regulate ribosome biogenesis and function and play diverse roles in gene expression regulation. The slight growth defect we observed suggested that METTL5 is likely not essential for ribosome biogenesis (21), but that it may regulate the translation of a subset of transcripts that collectively slows cell proliferation. Consistent with this, polysome profiling in both HeLa (Fig. 3, *B* and *C*) and HepG2 cells (Fig. S3*B*) shows no notable changes in global translation. We note that the effect of METTL5 depletion on global translation has differed across recently published reports on METTL5 function, especially across different cell lines (21, 22, 23, 24, 25).

To identify specific transcripts whose regulation may be disrupted by loss of METTL5, we sequenced ribosome-protected fragments (Table S5). At the global level, ribosome profiling experiments in HepG2 cells revealed more significant changes at the level of transcript translation than transcript abundance. In comparing METTL5-WT *versus* METTL5-KO cells, translation efficiency (TE) showed greater variance than transcript abundance (measured as reads per kilobase of transcript per million mapped reads, RPKM), suggesting that gene expression changes in METTL5-KO cells may primarily occur during transcript translation rather than transcription (Fig. 3*D*). This experiment also revealed several differentially translated transcripts in METTL5-KO cells (Fig. 3*E*), including not only METTL5 as expected, but also CALM1, a calcium-binding protein known to regulate cell proliferation and growth. Upon inspecting ribosome-protected fragment and input reads aligned to CALM1 in Integrated Genomics Viewer, we noted similar transcript levels in both METTL5-WT and METTL5-KO cells, but greatly increased ribosome occupancy in METTL5-KO cells, especially in exon 4 (Fig. S3*C*). In addition, gene ontology analysis revealed translational upregulation of genes related to mitochondrial biogenesis and regulation, detoxification, and reduction/oxidation processes (Fig. 3*F*), but it is not clear whether translation upregulation of these transcripts is directly due to the loss of 18S m^6^A or a compensation for broader cellular changes due to METTL5 loss. Overall, although our ribosome profiling data showed that a subset of transcripts was affected more than others, it did not point to a clear mechanism explaining the preference for certain transcripts. Additionally, while there was slight dysregulation across codons due to METTL5 depletion, no single codon or amino acid stood out as being particularly affected in terms of occupancy at the P-site, which is near 18S m^6^A1832 (Fig. S3*D*).

### *Mettl5*^*−*/*−*^ mice demonstrate growth and metabolic changes

**Figure 4:**
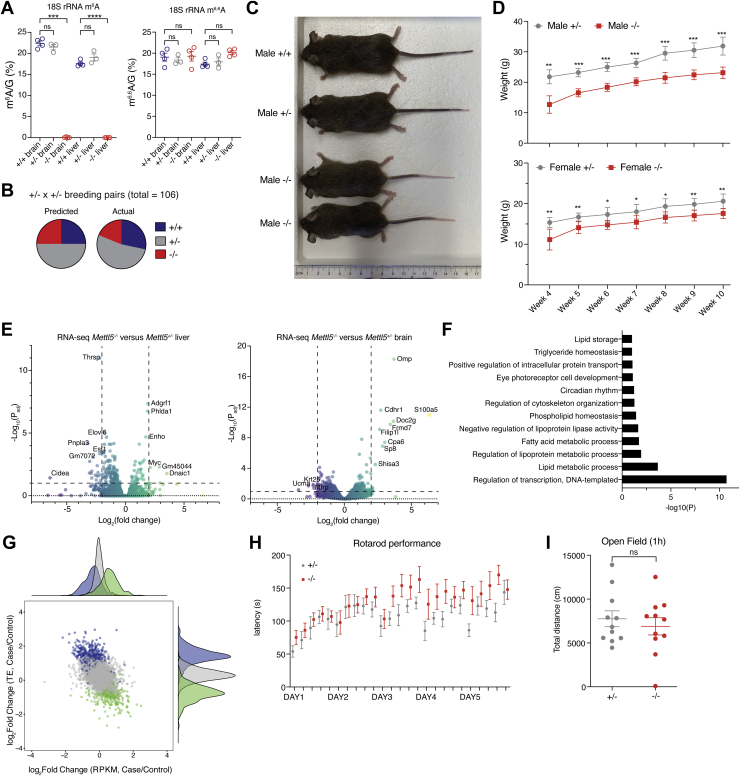
***Mettl5* knockout mice have developmental and metabolic defects.***A*, levels of m^6^A (*left*) and m^6,6^A (*right*), normalized to G, obtained by LC-MS/MS of 40-nt probe-purified segments of 18S rRNA surrounding the m^6^A 1832 site from the brains and livers of *Mettl5*^*+/+*^, *Mettl5*^+/−^, and *Mettl5*^*−/−*^ mice. n = 4, *Mettl5*^*+/+*^, n = 3, *Mettl5*^+/−^, and n = 4, *Mettl5*^*−/−*^. Analyzed by one-way ANOVA comparison with Sidak test for multiple comparisons. *B*, pie chart of the predicted (*left*) and actual (*right*) mice born of wild type (+/+, *dark blue*), heterozygous (+/−, *grey*), and knockout (−/−, *red*) genotypes from heterozygous x heterozygous breeding pairs (total = 106 mice; 30 +/+, 56 +/−, 20 −/− mice). *C*, photograph of male littermate *Mettl5*^*+/+*^, *Mettl5*^+/−^, and *Mettl5*^*−/−*^ mice at 8 weeks of age. *D*, body weights in grams of male (*top*) and female (*bottom*) *Mettl5*^+/−^ and *Mettl5*^*−/−*^ mice from 4 weeks (weaning) to 10 weeks of age. n = 6 mice for male +/−, n = 4 for male −/−, n = 5 for female +/− and −/−; unpaired *t* test was used to compare each time point. *E*, volcano plots from mouse liver (*left*) and brain (*right*) RNA-seq data displaying the negative log_10_ of the adjusted *p* value *versus* the log_2_ fold change comparing the transcriptomes of *Mettl5*^*−/−*^ and *Mettl5*^+/−^ mice (n = 3 pairs). *F*, dysregulated pathways based on analysis of RNA-seq data from *Mettl5*^*−/−*^ and *Mettl5*^+/−^ mouse livers by the software DAVID (65). *G*, effects of METTL5 knockout on translation and transcription as displayed by log_2_(fold change) of translation efficiency (TE) versus log_2_(fold change) of reads per kilobase of transcript per million mapped reads (RPKM) from ribosome profiling input and ribosome-protected fragment sequencing of mouse livers. Case = *Mettl5*^*−/−*^, Control = *Mettl5*^*+/+*^. *H*, latency in seconds for *Mettl5*^+/−^ and *Mettl5*^*−/−*^ mice to fall off the rotarod in a rotarod performance test. *I*, total distance in centimeters travelled by *Mettl5*^+/−^ and *Mettl5*^*−/−*^ mice when placed in a new environment in an open field test. Unpaired *t* test shown. *H* and *I*, n = 11 pairs. ns: not significant, ∗*p* < 0.05, ∗∗*p* < 0.01, ∗∗∗*p* < 0.005.

To assess METTL5 function at the level of a whole organism, we generated *Mettl5* knockout mice (*Mettl5*^*−*/*−*^) by disrupting exon 2 of *Mettl5* with CRISPR-Cas9 (Fig. S4*A*). *Mettl5* expression was undetectable in these mice by qPCR and greatly diminished by RNA-seq (Fig. S4, *B* and *C* and Table S6). Critically, m^6^A levels on 18S rRNA isolated directly from the brains and livers of *Mettl5*^*−*/*−*^ mice were abolished, while m^6^A levels in *Mettl5*^+/+^ and *Mettl5*^+/−^ mice were similar, and the neighboring m^6,6^A site remained stable, consistent with our findings in METTL5-KO HeLa and HepG2 cells (Fig. 4*A*). In agreement with Ignatova *et al.* (22), we observed that *Mettl5*^*−*/*−*^ mice are subviable and noted fewer than expected *Mettl5*^*−*/*−*^ mice born from both HET/HET (Fig. 4*B*) and HET/KO (Fig. S4*D*) breeding pairs. It was also visibly apparent that *Mettl5*^*−*/*−*^ mice were consistently smaller than *Mettl5*^+/+^ and *Mettl5*^+/−^ littermates (Fig. 4*C*). Monitoring mouse weight across several weeks revealed that this size difference persisted over time in both male and female mice, with *Mettl5*^*−*/*−*^ mice consistently weighing less than *Mettl5*^+/−^ mice (Fig. 4*D*). Though these measurements were taken from weeks 4 to 10, we noted that the difference was already present at the point of weaning (4 weeks) and persisted with similar magnitude, suggesting that this difference arose early in development, possibly even prior to birth.

In dissecting mice to harvest tissues for gene expression analysis, we noted that *Mettl5*^*−*/*−*^ mice consistently had less body fat than *Mettl5*^+/−^ or *Mettl5*^+/+^ mice (Fig. S4*E*). RNA-seq analysis of brain and liver tissues revealed gene expression patterns that suggest altered metabolism (Table S6). In particular, gene ontology analysis of genes downregulated in *Mettl5*^*−*/*−*^ mouse liver tissue revealed changes in genes involved in lipid biosynthesis and storage, consistent with the reduced body fat observed in *Mettl5*^*−*/*−*^ mice (Fig. 4, *E* and *F*). Noting the dramatic downregulation of *Thyroid Hormone Responsive Protein* (*Thrsp*) gene expression (Fig. 4*E*), we measured T3 thyroid hormone levels in blood collected from the mice used for these RNA-seq studies and found that T3 levels may be elevated in the blood of *Mettl5*^*−*/*−*^ mice relative to heterozygotes (Fig. S4*F*). Furthermore, we performed ribosome profiling and sequenced ribosome protected fragments in *Mettl5*^*+/+*^ and *Mettl5*^*−/−*^ mouse liver tissues to assess changes in translation (Table S7). Consistent with what we observed in METTL5-KO cell lines, while no significant changes in global translation were observed between polysome profiles from livers of *Mettl5*^*+/+*^ and *Mettl5*^*−/−*^ littermate pairs (Fig. S4*G*), TE showed greater variance than transcript abundance in our ribo-seq analysis (Fig. 4*G*). These data suggest that gene expression changes in *Mettl5*^*−/−*^ mice occur primarily at the level of translation. However, using the same parameters used to assess differentially translated genes in knockout cell lines (Figs. 3*E* and S4, *H* and *I*), we found there were few statistically significant differentially translated transcripts in *Mettl5*^*−/−*^ mouse livers compared with *Mettl5*^*+/+*^ littermate controls, potentially due to greater variability among mice (Fig. S4, *H* and *I*).

Since METTL5 mutations are associated with intellectual disabilities in human patients, we wanted to determine whether cognitive or behavioral changes were evident in our mouse model. Recent reports suggest that loss of *Mettl5* results in reduced locomotor activity and exploratory activity and defects in learning and memory (22, 27). However, in our mouse model, we did not observe similar defects in locomotor and exploratory activity from rotarod performance and open field tests (Fig. 4, *H* and *I*), in fear-based learning from a shuttle box test (Fig. S4*J*), or in instrumental learning from FR1 acquisition (Fig. S4, *K* and *L*). We note that our experiments were done comparing knockout mice with heterozygotes, not wild-type mice as in other studies. However, given that *Mettl5*^*+/+*^ and *Mettl5*^+/−^ mice have similar levels of 18S rRNA methylation (Fig. 4*A*), we believe that any differences between these mice are due to something other than differences in 18S rRNA methylation status. However, combined with small variations in experimental setup, the comparison of *Mettl5*^*−/−*^ to *Mettl5*^*+/+*^ *versus Mettl5*^+/−^ could contribute to the differences in outcomes in these behavior assays.

## Discussion

In our study, we identified TRMT112 as the primary binding partner of METTL5, a finding consistent with other recent reports (21, 22, 23). We found that protein–protein interactions between METTL5 and TRMT112 are important for METTL5 stability (Figs. 1, *D* and *E* and S1, *C* and *D*), and methyltransferase activity (Fig. 2*G*), though the latter is likely a consequence of the stabilizing effect of TRMT112 on METTL5. Of clinical significance, we found that the METTL5-TRMT112 interaction was severely reduced by mutations known to cause intellectual disability in humans (Fig. 1*C*) (18, 20). Structural analysis of the three major human variants reported by Richard *et al.* and Hu *et al.* (18, 20) suggests that all three mutations (R115Nfs∗19, K191Vfs∗10, G61D) would likely disrupt proper folding (Fig. S1*C*). Of these, the frameshift variants, R115Nfs∗19 and K191Vfs∗10, were demonstrated to cause lower METTL5 expression (18, 20). Structural analysis suggests that the METTL5^G61D^-TRMT112 complexes that do form may lack catalytic activity, since the G61D mutation creates a polar interaction with S-adenosyl methionine (Fig. S1*C*). Disruption of the METTL5-TRMT112 interaction likely also decreases the stability of these METTL5 variants, negatively impacting their methyltransferase activity and contributing to disease. To our knowledge, this is the first time that the METTL5-TRMT112 interaction has been investigated in the context of human disease. Intriguingly, our results further suggest the possibility that there are other pathways dysregulated by disease-causing METTL5 variants. For example, the availability of TRMT112 to its other binding partners, which include AlkBH8 and HemK2, could also be dysregulated in this context (29, 30, 34).

We also characterized METTL5 m^6^A methyltransferase activity and investigated its substrates *in vitro* and *in vivo*, identifying 18S rRNA m^6^A1832 as a major METTL5 substrate in mammalian cells, a result corroborated by recent reports (21, 22, 35). Interestingly, though the nucleolar localization of METTL5 pointed to its role in rRNA methylation, we also found significant cytoplasmic localization of METTL5 by fluorescence microscopy and biochemical fractionation (Fig. 2, *A* and *B*). Significant cytoplasmic localization has also been reported in neurons (20) and *Drosophila melanogaster* (23), raising questions about its cytoplasmic function. One plausible cytoplasmic role for METTL5 is late-stage methylation of rRNA, in line with analysis by van Tran *et al.* that found METTL5-TRMT112 complex near helix 44 only at late stages of processing (21, 36). METTL5 may also remain associated with the ribosome in the cytoplasm, where it could regulate translation by recruiting factors to the ribosome. We note, however, that Western blotting for METTL5 across polysome profiling fractions did not reveal an interaction with translating ribosomes in HeLa cells (Fig. 3*C*). Additionally, cytoplasmic METTL5 localization could suggest the existence of other METTL5 methylation targets, and differences in abundance and localization of substrates in various cell lines could contribute to discrepancies in its localization. Our CLIP-seq and m^6^A-seq identified a small subset of transcripts that were both associated with METTL5 and whose m^6^A levels changed in METTL5-KO cells, respectively, supporting this idea (Fig. S2, *A*–*D*). While some of these may represent indirect interactions, it is interesting that motifs mimicking the 18S m^6^A1832 sequence context are enriched in differentially methylated m^6^A peaks on transcripts overrepresented in the METTL5 CLIP data (Fig. S2*D*). Moreover, there is discordance among existing studies about non-18S rRNA substrates. While Ignatova *et al.* demonstrated METTL5 activity on polyA-enriched RNA, van Tran *et al.* did not identify any non-rRNA sites in their miCLIP screen (21, 22). This may be explained by the fact that in an *in vitro* experiment, in the absence of rRNA, activity is more likely detectable on other substrates such as polyA-enriched RNA. On the other hand, a miCLIP experiment could also miss more transient interactions on less abundant RNAs such as mRNAs. Altogether, existing data make it difficult to draw conclusions as to other possible METTL5 substrates, and this question warrants future investigation.

METTL5 activity on 18S rRNA prompted us to characterize the effects of METTL5 depletion on translation. Though polysome profiling did not reveal global translational changes in HeLa or HepG2 METTL5-KO cells (Figs. 3, *B* and *C* and S3*B*) or in *Mettl5*^*−/−*^ mice (Fig. S4*G*), ribosome profiling revealed dysregulated transcripts in cell lines (Figs. 3*E* and S4*H*) and greater variation at the translational than the transcriptional level (Figs. 3*D* and 4*G*). These data are consistent with 18S rRNA being a major cellular substrate of METTL5. Interestingly, a more drastic effect on polysomes has been documented in mouse embryonic stem cells (22, 24, 35). Clues to how METTL5 causes transcript- and context-specific effects may come from the location of the 18S m^6^A1832 site at the tip of helix 44 near the decoding center (4, 21). Rong *et al.* proposed that the methyl group may fine-tune the conformation of the decoding center and suggested that the methylated adenine and its base-pairing partner are in closer proximity to mRNA in the human ribosome than in structures lacking the methylation from other organisms (24). The location of m^6^A1832 on the 3′ end of 18S rRNA suggests that it could be surface exposed in some contexts and in close proximity to the mRNA being translated. The presence or absence of m^6^A1832 could thereby alter interactions between the mRNA and the ribosome, possibly even between 18S rRNA and the mRNA directly, thereby modulating translation efficiency. While we and others have observed through ribosome profiling that certain transcripts and codons are more affected than others (Figs. 3*E*, S3*D* and S4*H*), the specific codons and transcripts affected are not consistent across datasets (22, 35). Loss of 18S m^6^A1832 may also affect the position of helix 44, which is located near binding sites for key initiation and reinitiation factors, including eIF1, eIF1A, DENR, and eIF2D (37, 38, 39). Indeed, Rong *et al.* reported altered binding of the initiation factors eIF3A and eIF4E to translating ribosomes and decreased phosphorylation of the translation initiation-related signaling protein RPS6 in METTL5-KO HEK293T and HeLa cells (24). It is thus possible that some of the effects of METTL5 may be mediated by changes in binding of initiation-related factors to the ribosome. Considering the larger context beyond the ribosome itself may also elucidate differences in the effects of METTL5 knockout on translation in different cell lines, as there are known differences in translation-related signaling pathways among cell lines, especially in stem cells (40).

Our findings, particularly from our *Mettl5* knockout mouse model, provide insight into hereditary METTL5-related diseases caused by human variants. To our knowledge, this is the third *Mettl5* knockout mouse model reported, but the first for which 18S m^6^A loss was validated (22, 27). Guided by the observations that *Mettl5*^*−*/*−*^ mice weighed less (Fig. 4*D*) and had decreased body fat (Fig. S4*E*), we further investigated the metabolism of the mice through RNA-seq of their livers. We found dysregulated lipid, lipoprotein, and fatty acid metabolic pathways in knockout mice (Fig. 4*F*) and changes in thyroid hormone signaling (Figs. 4*E* and S4*F*). Interestingly, metabolic dysregulation was also suggested in a report of *mettl-5* knockout in *C. elegans* (25). The mechanisms leading to these changes remain unclear but could have clinical significance, as many patients with METTL5-associated microcephaly and intellectual disability reported by Richard *et al.* are reported to have reduced body weight (20). Unlike recent reports (22, 27), however, tests with our mouse model for neurological and behavioral deficits failed to show statistically significant differences between heterozygous and knockout mice in learning ability, motor activity, or exploratory activity (Figs. 4, *H* and *I* and S4, *J*–*L*), potentially due to differences in experimental setup or mouse model design (Fig. S4*A*). Another possibility is that the loss of activity in heterozygotes may be enough to affect neurological function, although the intellectual disability and microcephaly clinical phenotypes were reported to be autosomal recessive (20). The 18S rRNA m^6^A levels were also similar in *Mettl5*^*+/+*^ *versus Mettl*^+/−^ mice (Fig. 4*A*). Thus, such an effect would likely be unrelated to the rRNA methyltransferase activity of METTL5-TRMT112. We also note that the patient-derived METTL5 mutants are expressed to some degree, meaning our complete knockout cell lines and mouse model do not entirely mimic the physiological conditions. It also remains possible that the neurological and behavioral defects caused by METTL5 mutations may be more subtle than is easily detectable by the simple tasks or sample sizes we tested but significant enough in disrupting complex tasks in humans to cause clinical intellectual disability.

Given that 18S m^6^A1832 is currently the only validated substrate of METTL5 and the effects of METTL5 knockout seem to be most significant at the translational level, questions arise about how losing a single methyl group in the context of the whole ribosome may lead to organism-level effects. Notably, abnormalities in development and metabolism have been found in characterized clinical ribosomopathies (41). Furthermore, the differences seen in the effects of METTL5 in different cell types and tissues mirror other ribosomopathies that are very tissue-specific (9). Since our findings and those reported by others have solidified the role of METTL5 in ribosome biogenesis and function, we suggest that diseases caused by METTL5 variants could represent a previously uncharacterized ribosomopathy. The clinical relevance of METTL5 underscores the importance of future investigation into the mechanisms by which mutations lead to these phenotypes. Such studies should include comprehensive identification and validation of METTL5 substrates alongside detailed investigations into protein translation and signaling pathway changes, which may in turn guide the development of novel clinical interventions.

## Experimental procedures

### Cell culture

FreeStyle 293-F cells were obtained from Thermo Fisher (R79007), and HeLa (CCL-2) and HepG2 (HB-8065) cells were obtained from ATCC. HeLa and HepG2 cells were cultured in Dulbecco’s Modified Eagle Medium supplemented with 10% fetal bovine serum, sodium pyruvate, and penicillin/streptomycin (Gibco). FreeStyle 293-F cells were grown in suspension in FreeStyle 293 Expression Medium (Gibco).

### Construction and validation of knockout cell lines

HeLa and HepG2 METTL5-KO cell lines were generated *via* CRISPR-Cas9-mediated gene disruption. Guides (see table below) were cloned into pSpCas9(BB)-SA-Puro (PX459) V2.0, a gift from Feng Zhang (Addgene plasmid # 62988; http://n2t.net/addgene:62988) (42). In total, 100 pmol of each guide and its reverse complement were phosphorylated with T4 polynucleotide kinase for 30 min at 37 °C and then annealed by incubating at 95 °C for 5 min and then ramping down to 25 °C at 5 °C per minute. Phosphorylated, annealed guides were ligated into BbsI-digested PX459 using T4 DNA ligase. Constructs were sequence verified prior to transfection into HeLa and HepG2 cells. Combinations of two constructs were used to generate deletions: PX459/METTL5-guide2 and PX459/METTL5-guide32 to generate a deletion across exon 3 and 4, and PX459/METTL5-guide2 and PX459/METTL5-guide3 to generate a deletion across exons 2, 3, and 4 (Fig. S1*A*). These combinations of plasmids were transfected into HeLa or HepG2 cells using Lipofectamine 2000 according to manufacturer instructions. After 48 h, media was changed to media containing 1 μg/ml of puromycin, and cells were allowed to grow for approximately a week, with media changes as needed as cell death progressed. Remaining cells were then trypsinized and diluted such that they could be plated at approximately one cell per well. Single cells were allowed to grow over the course of 2 to 4 weeks and collected and expanded as needed. METTL5 KO cell lines were identified *via* PCR to verify the appropriate deletion and verified by Western blot.

**Table:** Guides

Guide	Sequence
M5 guide 2	CACCGAATTACTGTATCGAATGACT
M5 guide 2-RC	AAACAGTCATTCGATACAGTAATTC
M5 guide 3	CACCGAGAGAGTCGCCTGCAACAAG
M5 guide 3-RC	AAACCTTGTTGCAGGCGACTCTCTC
M5 guide 32	CACCGCAATGTCATCATAAGTGTTA
M5 guide 32-RC	AAACTAACACTTATGATGACATTGC

### DNA constructs

METTL5 coding sequence was amplified from pCMV-Entry-METTL5-Myc-DDK (Origene cat# PS100001) by PCR. The PCR product and pCS2-FLAG (a gift from Peter Klein, AddGene plasmid #16331; http://n2t.net/addgene:16331; RRID:Addgene 16331) were digested with EcoRI and XhoI and ligated with T4 DNA Ligase. Point mutations were made using quickchange mutagenesis with primers overlapping mutated sequences and encoding the following mutations: amino acids 127 to 129 NPP > AAA, G61D, and K191Vfs∗10delAA.

### FLAG-METTL5 coimmunoprecipitations

HeLa or Freestyle 293-F cells were transfected with constructs encoding FLAG-tagged METTL5 (WT or mutant) and were pelleted in cold 1x PBS by centrifugation at 1000*g* for 10 min at 4 °C. Cells were lysed in five pellet volumes of lysis buffer containing 50 mM Tris pH 7.4, 150 mM NaCl, 10% glycerol, 1.5 mM MgCl_2_, 0.5 mM EDTA, 0.2% NP-40, 1 mM DTT, and 1x SigmaFast protease inhibitor cocktail (Sigma) and rotated for 1 h at 4 °C. The lysate was clarified by centrifugation at 20,000g for 20 min at 4 °C. For immunoprecipitation, 30 μl FLAG-M2 beads per 10 million cells transfected were washed into lysis buffer three times before adding to clarified lysate. The mixture of lysate and beads was rotated for 1 h at 4 °C and then washed three times with 0.5 ml of lysis buffer each time. After the final wash, 2x SDS with 100 mM DTT was added to the beads and bound protein was eluted by heating at 65 °C for 15 min, shaking at 1000 rpm. Input and IP samples were separated on a 4 to 12% bis-tris gradient gel for Western blotting (below).

### Western blots

Cells were scraped into ice-cold PBS and pelleted by centrifugation at 1000*g* for 10 min at 4 °C. Cell pellets were resuspended in three pellet volumes of lysis buffer containing 50 mM Tris pH 7.4, 300 mM NaCl, 1% NP-40, 0.25% deoxycholate, 1 mM DTT, and 1x SigmaFast protease inhibitor cocktail (Sigma). Lysates were kept on ice for 1 hour with periodic agitation and then clarified by centrifugation at 20,000g for 20 min at 4 °C. Protein concentrations were measured by bicinchoninic acid (BCA) assay (Pierce) for normalization. Protein samples were separated on 4 to 12% bis-tris gradient gels using MOPS (Thermo Fisher) or MES (Thermo Fisher) buffer and then transferred to nitrocellulose membrane by semidry transfer at 17V for 50 min using 2x NuPage Transfer Buffer (Thermo Fisher) with 10% methanol. Membranes were blocked with 5% dry milk in 1x TBST (1x TBS with 0.1% Tween-20) for 30 min. Primary and secondary antibodies were diluted in 5% milk in TBST and incubated as indicated in the antibodies table (below). Membranes were imaged using a FluorChem R (ProteinSimple) or a ChemiDocMP (BioRad), and image analysis was performed with Fiji (43). Annotated, full scans of all Western blots are provided in Fig. S5.

### RNA purification

Total RNA was isolated from cells using Trizol (Invitrogen) according to the manufacturer’s instructions. After collecting cells in Trizol, 0.2 ml chloroform per 1 ml Trizol was added, and the samples were shaken vigorously for 15 s prior to incubating at room temperature for 2 min. Samples were then phase separated by centrifugation at 12,000g for 20 min at 4 °C. The aqueous phase was collected, mixed with isopropanol (0.5 ml per 1 ml Trizol used), incubated at room temperature (RT) for 10 min, and RNA was pelleted by centrifugation at 20,000g for 20 min at 4 °C. The pellet was washed with 80% ethanol, centrifuged at 10,000g for 10 min at 4 °C, and then dried and resuspended in RNase free water. Polyadenylated RNA was isolated using the Dynabeads mRNA Direct kit (Life Technologies) as per manufacturer instructions. Ribosomal RNA was depleted using the RiboMinus Eukaryote Kit v2 (Life Technologies) as per manufacturer instructions.

### siRNA transfection

One day prior to transfection, 150,000 HeLa cells were plated per 6 cm cell culture dish in antibiotic-free cell culture media. Media was changed to fresh antibiotic-free media just prior to transfection with 20 pmol siRNA and Lipofectamine RNAiMAX transfection reagent (Invitrogen). Forty-eight hours after transfection, cells were harvested either in Trizol (for qPCR analysis) or in ice-cold PBS (for Western blot analysis).

**Table:** siRNAs used:

Target/label	Source	Cat number	Sequence (sense)
Cont. #1	Sigma	SIC001–10NMOL	Proprietary sequence - siRNA Universal Negative Control #1
Cont. #2	Sigma	SIC002–10NMOL	Proprietary sequence - siRNA Universal Negative Control #2
METTL5 #1	Sigma	SASI_Hs01_00170949	CCAAGUCAUUCGAUACAGU[dT][dT]
METTL5 #2	Sigma	SASI_Hs01_00170950	GAAGAGUUUGAGUUAACAA[dT][dT]
TRMT112 #1	Sigma	SASI_Hs01_00034520	CCGAUAACUUGCGUCUGAU[dT][dT]
TRMT112 #2	Sigma	SASI_Hs01_00034522	CGGCCGAUAACUUGCGUCU[dT][dT]
TRMT112 #3	Sigma	SASI_Hs01_00034521	GUCUGAUCCAGGUGCCGAA[dT][dT]

### *In vitro* methyltransferase assays

Reactions were performed by combining the desired amounts of protein and total RNA or probe in 1x methyltransferase buffer (10 mM HEPES pH 7.4, 4% glycerol, 100 mM KCl, 1.5 mM MgCl_2_, 1 mM DTT), with 2 mM d_3_SAM (CDN Isotopes) and 1 mM (2% v/v) SUPERaseIN RNase inhibitor (Invitrogen), for a final volume of 50 μl. Reactions were incubated at 37 °C for 1 h before being snap frozen in liquid nitrogen and stored at −80 °C. Prior to LC-MS/MS analysis, samples were thawed, supplemented with 1 mM EDTA, and treated with Proteinase K (Sigma) at 37 °C for 1 h. Samples were purified by phenol/chloroform extraction and RNA precipitation (total RNA) or MyOne Streptavidin C1 beads (biotinylated probes; Invitrogen). For total RNA purification, an equal volume of acid phenol chloroform pH 4.5 (Invitrogen) was added to each sample, and the mixture was mixed for 2 minutes and allowed to sit 2 min at RT. After centrifugation at 16,000g for 15 min at 4 °C, the aqueous layer was transferred to a new tube. In total, 10% of the volume of 3 M sodium acetate pH 5.5 and three volumes of 100% ethanol were added, before mixing and incubating at −80 °C for 3 h. RNA was pelleted by centrifugation at 20,000g for 20 min at 4 °C, and then the pellets were washed with 80% ethanol and resuspended in nuclease-free water. For probe purification, each sample was diluted into 350 uL of 1x IP buffer (50 mM Tris pH 7.4, 300 mM NaCl, 0.5 mM EDTA, 1% v/v SUPERaseIN RNase inhibitor). Streptavidin C1 beads were prewashed three times in 1x IP buffer, and 10 μl bead resuspension was added to each sample. Samples were rotated for 2 h at 4 °C, washed in 1x IP buffer once, and then eluted with Trizol by shaking at RT for 20 min. RNA was purified from Trizol using the DirectZol RNA Miniprep kit (Zymo Research).

### LC-MS/MS

For each sample, 25 to 50 ng of RNA was digested with 1 μl Nuclease P1 (Sigma) in P1 buffer (25 mM NaCl, 2.5 mM ZnCl_2_) in a final reaction volume of 20 μl for 2 h at 42 °C. Subsequently, 1 μl of FastAP (Thermo Fisher) and 2.5 μl of 10x FastAP buffer were added to each sample, and they were incubated at 37 °C for 4 h. Samples were then diluted with an equal volume of water and filtered through a 0.2 μm PVDF filter (0.2 μm pore size, 0.4 mm diameter, Millipore). Five microliters of each filtered sample was separated by reverse-phase ultraperformance liquid chromatography on a ZORBAX Eclipse XDB-C18 Rapid Resolution HT 2.1 × 50 mm, 1.8 μm column (Agilent) on an Agilent Technologies 1290 Infinity II liquid chromatography system, followed by mass spectrometry on a Sciex Triple Quad 6500 triple-quadrupole mass spectrometer in positive electrospray ionization mode. Nucleosides were quantified using nucleoside-to-base transitions of 282.101 > 150.100 (m^6^A), 282.101 > 150.100 (m^1^A), 284.982 > 153.100 (d_3_m^6^A), 284.983 > 153.200 (d_3_m^1^A), 296.101 > 164.100 (m^6,6^A), 267.966 > 136.000 (A), and 284.004 > 152.100 (G). Standard curves were generated by injecting known concentrations of the corresponding pure nucleosides in the same run, and the percentage of modified to unmodified nucleosides was calculated based on the calibrated concentrations.

### Quantitative PCR (qPCR)

cDNA synthesis was performed in 20 μl reactions consisting of 1 μl random hexamer primers at 50 ng/μl (Invitrogen), 1 μl 10 mM dNTPs (NEB), 1 μl 100 mM DTT (Invitrogen), 1 μl SUPERaseIN inhibitor (Invitrogen), 1 μl Superscript IV reverse transcriptase (Invitrogen), 4 μl 5x SSIV buffer, and nuclease-free water. Samples were incubated in a thermocycler at 23 °C for 10 min, 55 °C for 10 min, and 80 °C for 10 min, then held at 4 °C. Ten microliter qPCR reactions were set up with 1 μl of cDNA, 5 μl FastStart Essential DNA Green Master mix (Roche), and 0.1 μl each of 10 μM forward and reverse primers (see table below). qPCR was performed on a LightCycler 96 (Roche) with the following settings: 95 °C, 600 s (preincubation); 95 °C 20 s, 60 °C 20 s, 72 °C 20 s (three-step amplification). Analysis was performed with LightCycler 96 SW1.1 software (Roche).

### Cell growth assays

To measure cell proliferation curves, four replicate 96-well plates were plated with four wells per cell line at 1000 cells per well (each plate represented one time point). For each time point, cell culture media was removed from each well, and the plate was placed directly into a −80 °C freezer for storage for the duration of the time course. The first time point was collected 3 h after plating (just enough time for cells to adhere to the plate surface) and used as time 0, to normalize for small differences in plated cell number. Subsequent time points were collected at 24, 48, and 96 h postplating and frozen at −80 °C for at least 24 h. The number of cells per well was quantified using the CyQuant Cell Proliferation Assay Kit (Invitrogen) as per the manufacturer’s instructions. Briefly, 200 μl of lysis buffer was added to each well, pipetted up and down 4 to 6 times and left at room temperature for 5 min. In total, 150 μl of this lysate was transferred to a black, clear-bottomed 96 well plate. Fluorescence was measured in a Synergy HTX plate reader (Biotek) using a 485/525 excitation/emission filter cube.

### METTL5 binding partner ID

Freestyle 293-F cells were seeded at 10^6^ cells/ml just prior to transfection with pCS2-FLAG-METTL5. Thirty-six hours after transfection, cells were pelleted by centrifugation at 500*g* for 5 min at 4 °C. The cells were washed with ice cold PBS and pelleted by centrifugation at 500*g* for 5 min at 4 °C. The washed cell pellet was lysed in five pellet volumes of lysis buffer (50 mM Tris pH 7.4, 50 mM NaCl, 10% glycerol, 1.5 mM MgCl_2_, 0.5 mM CaCl_2_, 0.5% NP-40, 1x SigmaFast protease inhibitor [Sigma], 1x PhosSTOP [Sigma], and 10units/ml DNaseI [NEB]) by rotating for 1 h at 4 °C. Lysate was first clarified by centrifugation at 10,000g for 10 min at 4 °C, and then the supernatant was transferred and clarified a second time by centrifugation at 20,000g for 20 min at 4 °C. In total, 500 μl FLAG-M2 magnetic beads (Sigma) were washed three times with 2 ml lysis buffer per wash and added to lysate and rotated for 30 min at 4 °C. After incubation with lysate, the beads were washed twice with 1.5 ml lysis buffer, three times with 1.5 ml high salt wash buffer (50 mM Tris pH 7.4, 500 mM KCl, 0.05% NP-40), and once with 1.5 ml PBS with 0.1% Tween-20. Bound proteins were then eluted with 500uL of 2x Laemmli sample buffer (BioRad) supplemented with 100 mM DTT by heating at 70 °C for 15 min, shaking at 1000 rpm. Samples were then separated on a 4 to 12% bis-tris gradient gel and stained by Silver Stain for Mass Spectrometry (Pierce). Bands of interest were excised using razor blades and stored in 1.5 ml tubes at −80 °C prior to being shipped on dry ice and analyzed by MS BioWorks, LLC (Band ID service).

To prepare samples, trypsin digestion was performed using a robot (ProGest, DigiLab) and processed as follows: samples were washed with 25 mM ammonium bicarbonate followed by acetonitrile, reduced with 10 mM dithiothreitol at 60 °C followed by alkylation with 50 mM iodoacetamide at RT, digested with trypsin (Promega) at 37 °C for 4 h, quenched with formic acid, and the supernatant was analyzed directly without further processing. The gel digest was analyzed by nano LC/MS/MS with a Waters NanoAcquity HPLC system interfaced to a Thermo Fisher Q Exactive. Peptides were loaded on a trapping column and eluted over a 75 μm analytical column at 350 nl/min; both columns were packed with Luna C18 resin (Phenomenex). The mass spectrometer was operated in data-dependent mode, with MS and MS/MS performed in the Orbitrap at 70,000 FWHM resolution and 17,500 FWHM resolution, respectively. The 15 most abundant ions were selected for MS/MS. Data were searched using a local copy of Mascot with the following parameters:

Enzyme: Trypsin

Database: Swissprot Human (forward and reverse appended with common contaminants)

Fixed modification: Carbamidomethyl

Variable modifications: Oxidation (M), Acetyl (Protein N-term), Pyro-Glu (N-term Q), Deamidation (NQ)

Mass values: Monoisotopic

Peptide Mass Tolerance: 10 ppm

Fragment Mass Tolerance: 0.02 Da

Max Missed Cleavages: 2

Mascot DAT files were parsed into the Scaffold software for validation, filtering and to create a nonredundant list per sample. Data were filtered using a minimum protein value of 90%, a minimum peptide value of 50% (Prophet scores) and requiring at least two unique peptides per protein.

### Fluorescence microscopy

In total, 20,000 cells were plated per acid-washed glass coverslip in a 24-well cell culture dish 24 h prior to fixation with 4% paraformaldehyde in PBS for 10 min at RT. Coverslips were then washed three times with PBS and transferred to a humidity chamber. Coverslips were incubated in blocking solution (1X PBS, 1% goat serum, 10 mg/ml BSA, 0.25% Triton X-100) for 30 min at RT, and then incubated with primary antibodies diluted in blocking buffer for 2 h at RT. After three 5 min washes with 1x PBS with 0.25% Triton X-100, coverslips were incubated with secondary antibodies diluted in blocking solution for 1 h at RT. After three 5 min washes with 1x PBS, the coverslips were mounted onto glass slides with ProLong Gold Antifade Mountant with DAPI (Life Technologies) and allowed to cure overnight at RT. Coverslips were imaged on an Olympus BX53 microscope with an Olympus U-HGLGPS light source using a 40X objective (NA 0.95). Antibodies used are listed in the table below.

### Cellular fractionation

Cellular fractionation was performed essentially as previously described (44). HeLa and HepG2 cells were scraped into ice-cold PBS and pelleted by centrifugation at 500*g* for 5 min at 4 °C. The cell pellet was gently resuspended and washed in five pellet volumes of Buffer A (10 mM HEPES pH 7.5, 4 mM MgCl_2_, 10 mM KCl, 10% glycerol, 340 mM sucrose, 1 mM DTT, 1x SigmaFast Protease Inhibitor [Sigma]) three times, pelleting cells by centrifugation 500*g* for 5 min at 4 °C between each wash. After the final wash, the pellet was resuspended in 2.5 pellet volumes of Buffer A. An equal volume of Buffer A + 0.2% Triton X-100 was added to this cell suspension with gentle mixing, and the samples then incubated on ice for 12 min with gentle inversion every 3 min. The lysate was then centrifuged 1200*g* for 5 min at 4 °C, pelleting nuclei. The supernatant (cytoplasmic extract) was transferred to a new tube, and nuclei washed three times with Buffer A + 0.1% Triton X-100, pelleting nuclei by centrifugation at 500*g* for 5 min at 4 °C between each wash and changing to fresh tubes once during these washes. The washed nuclear pellet was resuspended in one original cell pellet volume of NRB buffer (20 mM HEPES pH 7.5, 75 mM NaCl, 0.5 mM EDTA, 50% glycerol, 1 mM DTT, 1x SigmaFast Protease inhibitor [Sigma]). An equal volume of NUN buffer (20 mM HEPES pH 7.5, 10 mM MgCl_2_, 300 mM NaCl, 0.2 mM EDTA, 1 M urea, 1% NP-40, 1 mM DTT, 1x SigmaFast Protease inhibitor [Sigma]) was gently added to the nuclear suspension and incubated on ice for 5 min, with periodic inversion. After centrifugation at 1200*g* for 5 min at 4 °C, the supernatant (nuclear extract) was transferred to a new tube, and chromatin pellet washed twice with a 1:1 mixture of NUN/NRB buffer with centrifugation at 5000*g* for 3 min at 4 °C between each wash. The pellet was then washed twice with Buffer A in the same manner and resuspended in Buffer A after the final wash. Cytoplasmic, nuclear, and chromatin extracts were then treated with Benzonase (Sigma) for 30 min at 37 °C before being separated by gel electrophoresis and analyzed by Western blot.

### Cross-linking-assisted immunoprecipitation of METTL5 and sequencing

#### Immunoprecipitation

For each replicate experiment, fourteen 15 cm plates of ∼70% confluent HeLa cells were transfected with 5 μg of pCS2-FLAG-METTL5 plasmid per plate, using Lipofectamine LTX Reagent (Invitrogen) as per manufacturer’s instructions. Twenty-four hours after transfection, the cells were treated with 100 μM 4-thiouridine (Sigma) overnight. Thirty-six hours after transfection, cells were washed with cold PBS and cross-linked twice with 150 mJ per cm^2^ 254 nM UV in a cross-linker (UVP CL-1000), with plates kept on ice for the duration. Cells were harvested by scraping in ice-cold PBS and pelleting cells by centrifugation at 1000*g* for 10 min at 4 °C. Cell pellets were resuspended in five pellet volumes of lysis buffer (50 mM Tris pH 7.4, 150 mM NaCl, 10% glycerol, 1.5 mM MgCl_2_, 0.5 mM EDTA, 0.5% NP-40, 1x SigmaFast protease inhibitor) and rotated for 1 h at 4 °C. The lysate was clarified by centrifugation at 20,000g for 20 min at 4 °C, and an aliquot of lysate transferred to a new tube as an input sample. In total, 200 μl FLAG-M2 beads (Sigma) per 10 ml lysate were washed into lysis buffer three times before being added to clarified lysate. The bead/lysate suspension was rotated for 2 h at 4 °C, after which the beads were washed once with 1 ml lysis buffer and transferred to new tubes, then three times with 1 ml high-salt buffer (50 mM Tris pH 7.4, 500 mM KCl, 0.05% NP-40), followed by two more 1 ml washes with lysis buffer. The beads were then washed once with 1 ml Proteinase K buffer (50 mM Tris pH 7.4, 75 mM NaCl, 6.25 mM EDTA, 1% SDS) before resuspending the beads in 450 μl of this buffer (25 μl of these beads were transferred to a new tube for analysis by Western blot). Proteinase K (Sigma) was preincubated at 37 °C for 30 min prior to use. Fifty microliters of Proteinase K was added to beads resuspended in Proteinase K buffer. Inputs were similarly treated with Proteinase K by adding 225 μl 2x Proteinase K buffer (100 mM Tris pH 7.4, 150 mM NaCl, 12.5 mM EDTA, 2% SDS) and 25 μl Proteinase K to 250 μl of input sample. Proteinase K treatment of both input and bead samples was done by incubating samples 50 °C for 1 h, shaking at 1000 rpm. Three sample volumes of Trizol were added to each, and RNA was purified using the DirectZol kit (Zymo) as per manufacturer’s instructions. A technical note: our efforts to perform these experiments with the RNase digestion that is usually performed to reduce background and more precisely map protein binding sites were unsuccessful (45), possibly due to the small footprint of METTL5 and/or the transient nature of interactions between RNAs and catalytic enzymes.

#### Preparation of sequencing libraries

Sequencing libraries were prepared using the TruSeq Stranded mRNA library preparation kit (Illumina), using 30 to 40 ng of RNA for each input and IP sample. Samples were sequenced in one lane of an SR50 flow cell on a HiSeq4000 (50 bp single-end reads).

#### Data analysis

Analysis was performed very similarly to what would be done for an RNA immunoprecipitation (IP) experiment to assess which RNA transcripts are bound to a protein of interest. Three replicate experiments were performed as described above, with corresponding input and IP samples for each replicate. Quality of.fastq files was checked using FastQC v0.11.5 (http://www.bioinformatics.babraham.ac.uk/projects/fastqc/). Adapters were trimmed using Cutadapt (46) and files were then aligned to the human genome hg38 using hisat2 v2.1.0 (47) in splice-aware mode, and resulting bam files were indexed and sorted with samtools version 1.7 (48). DESeq2 (49) and R version 4.0.3 (50) were used to analyze differentially expressed transcripts, thereby identifying transcripts that were enriched in the IP samples relative to input samples.

### rRNA fragment purification

A 40-nt rRNA fragment was purified similarly to previously described (31). Briefly, 3 to 4 μg of a biotinylated DNA probe designed to bind to the target rRNA region was combined with 33 μg of total RNA in 3.33x hybridization buffer (250 mM HEPES pH 7, 500 mM KCl) in a total volume of 150 μl. The mixture was incubated at 90 °C for 7 min and then allowed to slowly cool to room temperature over 3.5 h to allow hybridization. Single-stranded RNA and DNA were digested by adding 0.5 μg RNase A (Thermo Scientific) to the mixture and incubating at 37 °C for 30 min, then 30 units of Mung Bean Nuclease were added along with 10x Mung Bean Nuclease buffer (NEB) to a final concentration of 1x, followed by a 30 min incubation at 37 °C. Streptavidin T1 beads (20 μl per sample; Invitrogen) were washed three times in 1 ml 2.5x IP buffer (375 mM NaCl, 125 mM Tris pH 7.9, 0.25% NP-40), then resuspended in 100 μl 2.5x IP buffer. The beads were combined with the 150 μl RNA-DNA hybridization mix and rotated at 4C for 1 h. After rotation, the beads were washed three times in 1x IP buffer (150 mM NaCl, 50 mM Tris pH 7.9, 0.1% NP-40), twice in nuclease-free water, and resuspended in 30 μl nuclease-free water. The resuspended beads were heated at 70 °C for 5 min to denature the RNA from the probes and quickly placed on ice before placing on a magnet to collect the RNA-containing supernatant.

### m^6^A-seq

#### Experiment

One microgram of 1x ribo-depleted RNA per sample (from HeLa METTL5-WT and METTL5-KO cells) was fragmented using the Bioruptor Pico sonicator (Diagenode) 30 s on, 30 s off, 30 cycles. About 1/20th of each sample was saved as input, and immunoprecipitation was performed using the EpiMark *N*^6^-methyladenosine Enrichment Kit (NEB) as per manufacturer’s instructions. Briefly, 1 μl m^6^A antibody per sample was coupled to prewashed Protein G Magnetic Beads (NEB) in 1x reaction buffer (150 mM NaCl, 10 mM Tris-HCl pH 7.5, 0.1% NP-40 in nuclease-free water) with orbital rotation for 30 min at 4 °C. Beads were washed, and fragmented RNA was added in 1x reaction buffer with 2% v/v SUPERaseIN RNase inhibitor (Invitrogen). Beads were washed twice each in 1x reaction buffer, low-salt reaction buffer (50 mM NaCl, 10 mM Tris-HCl pH 7.5, 0.1% NP-40 in nuclease-free water), and high-salt reaction buffer (500 mM NaCl, 10 mM Tris-HCl pH 7.5, 0.1% NP-40 in nuclease-free water), then resuspended at room temperature in 30 μl Buffer RLT (Qiagen) per sample. Eluates were purified using the RNA Clean & Concentrator-5 kit (Zymo Research). Libraries were then constructed using the SMARTer Stranded Total RNA-Seq Kit v2 (Takara) per manufacturer’s instructions and pooled and sequenced evenly across one lane of an Illumina NovaSeq6000 SP flow cell with 50 bp paired-end reads.

#### Data analysis

Quality of fastq files was checked using FastQC v0.11.5 (http://www.bioinformatics.babraham.ac.uk/projects/fastqc/). As instructed by the SMARTer Stranded Total RNA-Seq Kit v2 manual, the three bases at the start of all R2 reads were removed using Trimmomatic 0.39 (51) with the option HEADCROP:3. Adapter trimming and quality filtering were also performed with Trimmomatic using paired-end mode with options LEADING:3, TRAILING:3, SLIDINGWINDOW: 4:15, and MINLEN: 21. To check for contamination, files were aligned to the *mycoplasma* genome using hisat2 v2.1.0 (47) and any matching reads were discarded. Files were then aligned to the human genome hg38 using hisat2 v2.1.0 in splice-aware paired-end mode, and resulting bam files were sorted with samtools version 1.7 (48). Using the MeRIPTools package (52), reads were counted with the function CountReads, and peaks were called using Fisher’s exact test with the function callPeakFisher. The R package QNB was used for inferential testing, and differentially methylated peaks were called with an adjusted *p* value <0.1 (53). Motif searches were performed using HOMER v4.11 (54). For a background reference, sequences were extracted from random 200 bp peaks that were sampled from an mRNA transcript (52).

### Polysome and ribosome profiling

Polysome profiling was performed similarly to previous reports (55, 56). Four 15 cm plates per sample were cultured to ∼80% confluence. Each plate was treated with cycloheximide at a final concentration of 100 μg/ml in antibiotic-free medium and incubated at 37 °C for 7 min. Plates were washed with 10 ml of ice-cold PBS with 100 μg/ml cycloheximide twice, then cells were collected in ice-cold PBS and centrifuged at 500g for 5 min. Each pellet was resuspended in three pellet volumes of lysis buffer (20 mM HEPES pH 7.6, 100 mM KCl, 5 mM MgCl2, 1% Triton x-100, 100 μg/ml cycloheximide, 1% v/v SUPERaseIN inhibitor in nuclease-free water), and cells were lysed with rotation at 4 °C for 20 min before being centrifuged at 16,000 g for 15 min. To the clarified lysate, 4 μl of Turbo DNase (Thermo Fisher) was added, and samples were incubated at room temperature 15 min and then centrifuged again to clear the lysate. The absorbance at 260 nm of each lysate was measured by Nanodrop (Thermo Fisher), and samples were adjusted to the same optical density with lysis buffer. One-fifth of each sample was saved as input, while the remaining portion of each sample was loaded on a 5 to 50% w/v sucrose gradient prepared in lysis buffer in a SETON Scientific open top polyclear tube (cat# 7042). Samples were then centrifuged at 28,000 rpm for 3 h at 4 °C using an Optima L-100XP centrifuge with an SW28 rotor. After centrifugation, absorbance was read, and fractions were collected by Gradient Station (BioComp) equipped with an ECONO UV monitor (BioRad) and fraction collector (Gilson). The max absorbance (AUFS) was set to 0.5, number of fractions to 30, and distance/fraction to 3.2 mm.

Ribosome profiling was performed similarly, with a few key differences. Firstly, after DNase treatment, about 20% of the sample was saved as input with three volumes of Trizol added, while the other 80% was treated with 3 μl MNase (NEB) in 1x MNase buffer for 15 min at room temperature to digest RNA not covered by ribosomes. Additional SUPERaseIN (Invitrogen) was added to a final concentration of 0.5 mM to quench the reaction. Then, samples were loaded on 5 to 50% w/v sucrose gradients and fractionated as above. Secondly, after fractions were collected, samples were processed for sequencing. Fractions containing the digested monosomes were collected and pooled, and three volumes of Trizol (Invitrogen) were added to each sample. RNA purification was performed for both input and ribosome-protected fragment (RPF) samples by chloroform extraction followed by isopropanol precipitation, and one round of rRNA depletion was performed (see “RNA purification” section). Input RNA was fragmented by combining 20 μl RNA with 7.5 μl PNK buffer A (Thermo Fisher) and incubating at 94 °C for 25 min, then end repaired by diluting in 37.5 μl nuclease-free water, adding 10 μl T4 PNK (Thermo Fisher) and incubating at 37 °C for 30 min. Then, 12 μl 10 mM ATP (EMD Millipore), 2 μl 10x PNK buffer A, and 6 μl T4 PNK were added to incubate at 37 °C for 30 min. These samples were purified using the RNA clean and concentrator-5 kit (Zymo Research). Purified RPF RNA was run in 1x Novex TBU sample buffer (Thermo Fisher) on a 10% TBE-urea gel (Thermo Fisher) in 1x RNase-free TBE buffer (Thermo Fisher) with an IDT 10/60 loading control (IDT) for 1 h at 180 V. The gel was stained 15 min in 1x SYBR gold in TBE with gentle shaking and visualized using a Gel Doc EZ Imager (Biorad). Fragments between ∼26 and 34 nucleotides (corresponding to the region between ∼20 and 30 nt of the IDT loading control) were excised and purified by Small-RNA PAGE recovery kit (Zymo Research) per manufacturer’s instructions. RPFs were end repaired similarly to inputs with T4 PNK. Input and RPF RNA samples were used to prepare libraries for sequencing using the NEBNext Small RNA Library Prep Kit (NEB) per manufacturer’s instructions and were sequenced by Illumina NovaSeq6000 with 100 bp single-end reads on two SP flowcells.

Ribosome and polysome profiling of mouse tissues was performed similarly to the method in cell lines, with a few specific changes in sample acquisition, cycloheximide treatment, and lysis (57). Mice were sacrificed and livers were removed and snap-frozen very quickly following standard procedures (57), then stored at −80 °C. Each tissue sample was ground to powder under liquid nitrogen by mortar and pestle, and the powder was transferred to a sterile 10 cm cell culture plate on dry ice. An appropriate volume (∼1 ml per ∼200 mg tissue) of lysis buffer containing cycloheximide (10 mM Tris-HCl pH 7.5, 100 mM KCl, 5 mM MgCl_2_, 0.5% w/v sodium deoxycholate, 1% v/v NP-40 alternative, 10 mM DTT, 150 μg/ml cycloheximide, 1 U/μl SUPERase In RNase inhibitor, and 1x EDTA-free Roche protease inhibitor cocktail in RNase-free water) was added to the powder sample immediately upon removal from dry ice to resuspend it, and the plate was placed on ice for 1 to 2 min. Each sample was resuspended by gently pipetting ten times before transferring it to a clean microcentrifuge tube and leaving on ice for 3 to 5 more minutes to ensure complete lysis. Samples were spun at 2000g for 5 min at 4 °C to pellet the nuclei and tissue debris. The supernatant was transferred to a fresh tube and spun at 12,000g for 5 min at 4 °C to pellet the remaining cellular debris. Again the supernatant was transferred to a fresh tube to start DNase treatment. From there, the same protocols used for polysome and/or ribosome profiling of cell lines were followed, except that the max absorbance (AUFS) was set to 1.0 during fraction collection. Mouse ribosome profiling libraries were sequenced by Illumina NovaSeq6000 with 50 bp paired-end reads on two SP flowcells.

#### Data analysis

Analysis of polysome profiles was performed using Microsoft Excel and GraphPad Prism. For ribosome profiling from cell lines, fastq files from the two flowcells were merged using the linux command “cat.” For all ribosome profiling sequencing data, data quality was checked with FastQC v0.11.5 (http://www.bioinformatics.babraham.ac.uk/projects/fastqc/). Adapters were trimmed and quality filtering was performed with BBDuk from BBTools version 38.84 using a reference fasta file of the NEBNext adapter sequences and settings ktrim = 4, qtrim = rl, trimq = 20, k = 31, mink = 11, hdist = 1, entropy = 0.5, entropywindow = 30, entropyk = 5, minlen = 25 and maxlen = 34 (for RPFs), and minlen = 15 (for inputs) (BBtools: (https://sourceforge.net/projects/bbmap/). For input files, alignment to human genome hg38 (58) was performed using STAR 2.7.3a (59) in quantMode TranscriptomeSAM. Bam files were sorted and uniquely mapped reads extracted with samtools v1.7 (48), followed by featureCounts v1.6.0 (60) to create a counts table from mapped reads with options -s 1, -g gene_id, and -t exon. rRNA reads were removed from RPF Fasta files using BBDuk with a reference fasta file of human rRNA sequences. Analysis was then performed on the RiboToolkit server (http://rnabioinfor.tch.harvard.edu/RiboToolkit/) (61). First, RPF files were uploaded for single-case submission in collapsed fasta format with the following options: species = homo sapiens (hg38), RPF interval = 25 to 34, allowed mismatch = 2, max multiple-mapping = 1, no duplicate removal, offsets to infer P-sites—calculate by RiboToolkit, min coverage for pause sites = 10, fold change for pause sites = 20, ORF *p*-value = 0.05. Input counts table and single case Job IDs were then used as inputs for group case analysis with F value = 2 and *p* value = 0.05.

Analysis was performed similarly for mouse liver ribosome profiling data except that alignments were performed to the mm10 genome (58), and changes were made to accommodate 50 bp paired-end data for inputs. For example, BBDuk and STAR 2.7.3a were run in paired-end mode with paired input files, and FeatureCounts was run using the flags -p and -countReadPairs. The STAR reference genome generated before mapping was made with sjdbOverhang of 49 (rather than 100) to accommodate the shorter reads. For RPF samples, only read 1 was used, and data were analyzed as above for cell line data. For RiboToolkit single-case submissions, mus musculus (mm10) was selected for the species.

### RNA-seq, METTL5-KO HeLa cells

#### Experiment

Three biological replicates each of wild type and two different clones METTL5-KO HeLa cells (clone 1 and 7) were grown to confluency prior to lysis in Trizol (Invitrogen). Total RNA was purified as described above (“RNA purification”), and sequencing libraries were generated using an HIV reverse transcriptase evolved for m^1^A detection, as previously described (62). Libraries were sequenced using an Illumina NovaSeq6000 on an S1 flowcell with 100 bp paired end reads. For RNA-seq analysis, only read 1 (R1) was used.

#### Analysis

Quality of fastq files was checked using FastQC v0.11.5 (http://www.bioinformatics.babraham.ac.uk/projects/fastqc/). Adapters were trimmed using Cutadapt (46). To check for contamination, files were aligned to the *mycoplasma* genome using hisat2 v2.1.0 (47) and any matching reads were discarded. Files were then aligned to the human genome hg19 (58) using hisat2 v2.1.0 (47) and resulting bam files were indexed and sorted with samtools version 1.7 (48). Differential expression analysis was performed using DESeq2 (49) and R version 4.0.3 (50). Gene ontology analysis was performed with MetaScape (63).

### RNA-seq, mouse tissues

#### Experiment

Mouse organs were collected immediately upon sacrifice, washed in ice-cold PBS, placed in approximately four volumes of Trizol (Invitrogen), and then sonicated using a handheld sonicator (OMNI International) with a few brief pulses until there were no more visible chunks. Samples were stored at −80 °C until use. Libraries were prepared using the SMARTer Stranded Total RNA-Seq Kit v2 (Takara) per manufacturer’s instructions and sequenced on a NovaSeq6000 with 100 bp single-end reads.

#### Data analysis

Sequencing data quality was checked by FastQC v0.11.5 (http://www.bioinformatics.babraham.ac.uk/projects/fastqc/). Adapter trimming and quality filtering were performed with Trimmomatic (51) in single-end mode with options LEADING:3, TRAILING:3, SLIDINGWINDOW:4:15, and MINLEN:36. Reads were then aligned to the mm10 genome (58) using STAR 2.7.3a (59). Read counts mapping to each gene were obtained using featureCounts (60) with options -s 2, -g gene_id, and -t exon. Differential expression analysis was then performed in R version 4.0.3 (50) using DESeq2 1.28.1 (49). Gene ontology analysis was performed with MetaScape (63).

### Knockout mouse generation and mouse husbandry

*Mettl5* knockout mice were generated at the Gene Targeting & Transgenic Facility at Janelia Research Campus. A *Mettl5* conditional knockout line was made in which exon 2 was floxed, such that presence of Cre would induce exon 2 deletion by creating a frame-shift mutation. The construct was electroporated with gRNA/Cas9 protein into ES cells that are F1 hybrid of C57bl/6 x 129S6. F1 mice were transferred from Janelia Research Campus to the University of Chicago where the second generation was backcrossed to the B6 strain. To generate a whole body knockout, the conditional knockout strain described above was then crossed with the B6.C-Tg(CMV-cre)1Cgn/J line (The Jackson Laboratory, 006054) (64). All mice were housed in a specific pathogen-free facility. Both male and female mice were used throughout the study, between 4 weeks and 6 months of age. All experiments were approved by the University of Chicago Institutional Animal Care and Use Committee.

### ELISA

Blood was collected retro-orbitally from mice upon sacrifice, allowed to coagulate at room temperature for about 1 hour, and centrifuged at 10,000g for 15 min at 4 °C. The supernatant (serum) was transferred to a new tube and stored at −80 °C. The thyroid hormone (T3) concentration was assayed using the Biomatik Mouse Tri-iodothyronine, T3 ELISA Kit (Cat# EKC40189), per manufacturer’s instructions.

### Mouse behavioral assays

#### Rotarod experiment

A computer-controlled rotarod apparatus (Rotamex-5, Columbus Instruments) with a rat rod (7 cm diameter) was set to accelerate from 4 to 40 revolutions per minute over 300 s, and time to fall was recorded. Mice received five consecutive trials per session, one session per day (about 30 s between trials).

#### Open field

Open-field chambers were 40 cm x 40 cm (Med Associates) with lighting at 21 lux. Infrared beams recorded the animals’ locomotor activity and rearing movements (vertical activity). Mice were put in the open fieled chamber for 1 h to record their activity.

#### Passive avoidance

The shuttle box used contained two chambers: one chamber illuminated and the other dark (Kinder Scientific). Mice were transported to the behavior room and were handled for 3 minutes for 3 days before the passive avoidance experiment. During tasks, the right chamber remained illuminated while the left chamber remained dark. Training began by placing the mouse into the illuminated chamber facing away from the shut guillotine door. The mouse was allowed to explore the illuminated chamber for 2 min. The door was then opened to let the mouse explore both the illuminated and dark chambers for 5 min. At the end of this exploration period, the door was shut after returning the mouse into the illuminated chamber. Two minutes later, the door was opened. Latency to step into dark chamber was recorded by the computer as the baseline. Upon entering the dark chamber, the door was closed and one foot shock (0.2 mA, 2 s) was delivered. Ten seconds later, the mouse was removed from the dark chamber and put back to the home cage. After 24 h, the mouse was put into the light chamber for 2 min, and then the latency to step into dark chamber was recorded as the 24 h memory.

#### FR1

Mice were food deprived each day before the experiment. Mice had unlimited food access for 3 h each day after the experiment. Each mouse was first trained in an illuminated operant box (Med Associates) on a FR1 schedule for 30 min each day for about 3 weeks, with additional random delivery of a food pellet for the first 15 min (one pellet per minute on average). The maximum number of food pellets by pressing the lever was set at 40. Mice were then trained on a FR1 schedule without free delivery of the food pellets. After the mice achieved more than 15 presses for two consecutive days, the results of the last of day FR1 were compared between genotypes.

### Statistical analyses

Statistical analyses were done in GraphPad Prism. Throughout the figures, replicates are plotted as individual points, along with mean and standard error of the mean (s.e.m.). For experiments comparing two conditions, unpaired *t* test was used. For experiments with multiple comparisons, one-way ANOVA was used to test specific comparisons as indicated (that is to say, not every single sample was compared with every other sample). A Dunnett’s test was used to correct for multiple comparisons when all comparisons were made to a single control (Figs. 1*F*, 2*E* and S2*G*). When multiple comparisons were made between experimentally determined pairs, the Sidak method was used to correct for multiple comparisons (Figs. 4*A* and S4*J*). In experiments with multiple variables and parameters tested, a two-way ANOVA with the appropriate multiple comparison correction was used (Sidak test for Fig. 2*F*, Tukey test for Fig. 2*G*).

### TMT11 proteomic analysis of biochemically active fractions

HeLa nuclear extract was fractionated into 11 fractions, and a few active fractions were identified. Each fraction was loaded on 10% SDS-PAGE gel and run a short time with little separation. The proteins were then in-gel digested and labeled with 11 different tandem mass tags (TMT; Thermo Scientific). The TMT-labeled peptides were pooled and separated into six fractions by an Ultra-micro spin C18 column by eluting with 10%, 20%, 25%, 30%, 35%, and 70% of buffer B respectively (buffer A: 10 mM NH_4_COOH (pH=8.0); buffer B: buffer A plus 90% acetonitrile). Every fraction was further separated on a reverse phase column (30 cm X 75 μm, 1.9 μm C18 resin) during a 4 h gradient of 12 to 36% buffer B (buffer A: 0.2% formic acid, 3% DMSO; buffer B: buffer A plus 65% acetonitrile) and analyzed by Q-Exactive HF (Thermo Scientific) with one MS scan and up to 20 data-dependent high-resolution MS/MS scans. The MS/MS raw files were processed using the JUMP searching engine against the UniProt human database. Searches were performed using 15 ppm mass tolerance and allowing up to two missed trypsin cleavage sites. TMT tags on lysine residues and peptide N termini (+229.162932 Da) and carbamidomethylation of cysteine residues (+57.021 Da) were used for static modifications, and the dynamic modifications included oxidation of methionine residues (+15.99492 Da). Proteins were quantified by summing reporter ion counts across all matched PSMs using our in-house software. The mass spectrometry proteomics data are available *via* the PRIDE database (http://www.proteomexchange.org) under accession number PXD028832.

**Table:** Table of primers and oligonucleotides

Description	Application	Sequence
Mouse Mettl5 FOR	qPCR	AGAACAGTATCCCACCAGGC
Mouse Mettl5 REV	qPCR	ATCCAACACACAACCCTGCT
Human METTL5 FOR	qPCR	GGGTTAGCCGGGAGATCCT
Human METTL5 REV	qPCR	GCAGGCGACTCTCTAGTTCC
Human TRMT112 FOR	qPCR	GGCCGATAACTTGCGTCTGA
Human TRMT112 REV	qPCR	GGGTGCCCTCTATCACTTCC
Human HPRT1 FOR	qPCR	TGACACTGGCAAAACAATGCA
Human HPRT1 REV	qPCR	GGTCCTTTTCACCAGCAAGCT
Mouse Hprt FOR	qPCR	CTGGTGAAAAGGACCTCTCGAAG
Mouse Hprt REV	qPCR	CCAGTTTCACTAATGACACAAACG
28S biotinyl. DNA probe (4200–4240)	rRNA fragment purif.	Biotin-CTCGCCTTAGGACACCTGCGTTACCGTTTGACAGGTGTAC
18S biotinyl. DNA probe (1821–1860)	rRNA fragment purif.	Biotin-TTCCGCAGGTTCACCTACGGAAACCTTGTTACGACTTTTA
28S rRNA 12mer	*In vitro* Mtase assay	CGGUAACGCAGG-biotin
18S rRNA 12mer	*In vitro* Mtase assay	UCGUAACAAGGU-biotin
18S rRNA 60mer	*In vitro* Mtase assay	UCUAGAGGAAGUAAAAGUCGUAACAAGGUUUCCG UAGGUGAACCUGCGGAAGGAUCAUUA-biotin

**Table:** Antibodies

Target	Source/catalog number	Application/species/dilution
anti-METTL5	ProteinTech/16791-1-AP	Western blot/rabbit/1:500 dilution
anti-METTL5	Atlas/SAB2101471	IF/rabbit/1:250 dilution
anti-TRMT112	Santa Cruz Biotechnology/sc-398481	Western blot/mouse/1:100 dilution
anti-FLAG-M2	Sigma/A8592	Western blot/mouse/1:10,000 dilution
anti-RPL4	Proteintech/11302-1-AP	Western blot/rabbit/1:1000 dilution
anti-RPS6	Abcam/70227	Western blot/rabbit/1:1000 dilution
anti-EIF3A	Cell Signaling Technology/3411	Western blot/rabbit/1:1000 dilution
anti-EIF4E	Cell Signaling Technology/2067	Western blot/rabbit/1:1000 dilution
anti-GAPDH-HRP	GenScript/A00192-100	Western blot/goat/1:10,000 dilution
anti-SNRP70	Abcam/ab83306	Western blot/rabbit/1:1000 dilution
anti-histone H3	Cell Signaling Technology/D1H2	Western blot/rabbit/1:2000 dilution
anti-fibrillarin	Abcam/ab4566	IF/mouse/1:250 dilution
goat-anti-rabbit-488	Thermo Fisher/A11008	IF secondary, 1:500 dilution
goat-anti-mouse-594	Thermo Fisher/A11005	IF secondary, 1:500 dilution
goat anti-rabbit HRP	Thermo Fisher/A16110	Western blot, secondary, 1:5000 dilution
goat anti-mouse HRP	Thermo Fisher/A16078	Western blot, secondary, 1:5000 dilution
donkey-anti-rabbit DyLight 800	Thermo Fisher/SA5-10044	Western blot, secondary, 1:5000 dilution
donkey-anti-rabbit DyLight 680	Thermo Fisher/SA5-10170	Western blot, secondary, 1:5000 dilution

## Data availability

Raw and processed data files from all high-throughput sequencing experiments have been deposited in the NCBI Gene Expression Omnibus (GEO) with the following accession numbers: GSE174435 (METTL5 CLIP-seq), GSE174503 (RNA-seq of HeLa cells), GSE174420 (m^6^A-seq), GSE174418 (RNA-seq of mouse tissues), GSE174419 (HepG2 ribosome profiling), GSE188798 (mouse ribosome profiling). The mass spectrometry proteomics data are available via the PRIDE database (http://www.proteomexchange.org) under accession numbers PXD028832 (TMT11) and PXD029574 (METTL5 IP).

## Supporting information

This article contains supporting information (21, 54, 61, 63, 66).

## Conflict of interest

C. H. is a scientific founder and a member of the scientific advisory board of Accent Therapeutics, Inc.

## Supporting information

Supplemental Table S8

Supplemental Figures S1–S6, Supplementary Note
