# Supplementary material for: The METTL5-TRMT112 N6-methyladenosine methyltransferase complex regulates mRNA translation via 18S rRNA methylation
Source: J Biol Chem. 2022 Jan 14;298(3):101590. doi: 10.1016/j.jbc.2022.101590 (PMC8857481; doi:10.1016/j.jbc.2022.101590)
Supplement: Supplemental Figures S1–S6, Supplementary Note [file mmc9.pdf]

Supporting information

The METTL5-TRMT112 N^6-methyladenosine methyltransferase complex regulates mRNA translation via 18S rRNA methylation

Caraline Sepich-Poore[1,2,3,5], Zhong Zheng[1,2], Emily Schmitt[1,2], Kailong Wen[4], Zijie Scott Zhang[1,2,9], Xiao-Long Cui[1,2], Qing Dai[1], Allen C. Zhu[1,2,3,5], Linda Zhang[1,2], Arantxa Sanchez Castillo[1,2], Haiyan Tan[6,7], Junmin Peng[6,7], Xiaoxi Zhuang[4], Chuan He[1,2,3,8]*, Sigrid Nachtergaele[1,2,10]*

[1]Department of Chemistry, [2]Institute for Biophysical Dynamics, [3]Department of Biochemistry and Molecular Biology, [4]Department of Neurobiology, University of Chicago, Chicago, IL USA; [5]University of Chicago Medical Scientist Training Program, Chicago, IL, USA; [6]Center for Proteomics and Metabolomics, St. Jude Children's Research Hospital, 38105, Memphis, TN, USA; [7]Departments of Structural Biology and Developmental Neurobiology, St. Jude Children's Research Hospital, 38105, Memphis, TN, USA [8]Howard Hughes Medical Institute, University of Chicago, Chicago, IL, USA; [9]current address: State Key Laboratory for Conservation and Utilization of Bio-resource and School of Life Sciences, Yunnan University, Kunming, China; [10]current address: Department of Molecular, Cellular, and Developmental Biology, Yale University, New Haven, CT, USA

<u>Supplorting information associated with this manuscript:</u>

Supplementary figures 1-6
Supplementary note: Biochemical screen that resulted in METTL5 identification
Table S1. Proteomics analysis of METTL5 binding partners
Table S2. Analysis of transcripts isolated from FLAG-METTL5 CLIP
Table S3. METTL5 knockout versus wild type HeLa m^{6}A-seq differentially methylated peaks
Table S4. Differentially expressed genes in HeLa-METTL5-KO cells relative to HeLa-WT cells
Table S5. METTL5 knockout versus wild type HepG2 ribosome profiling data
Table S6. METTL5 knockout versus wild type mouse RNA-seq data
Table S7. *Mettl5*$^{-/-}$ versus *Mettl5*$^{+/+}$ mouse ribosome profiling data
Table S8. Total proteins quantified by TMT11-LC-MS/MS

Supplementary Figure 1

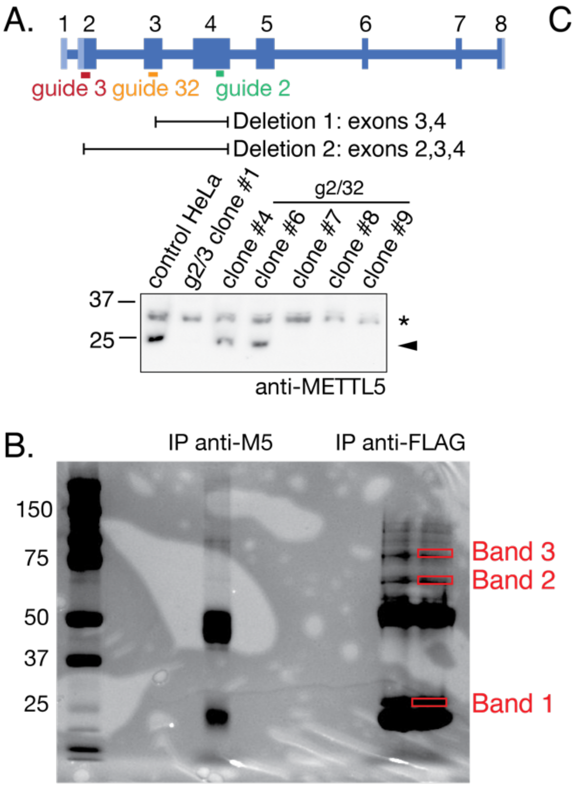

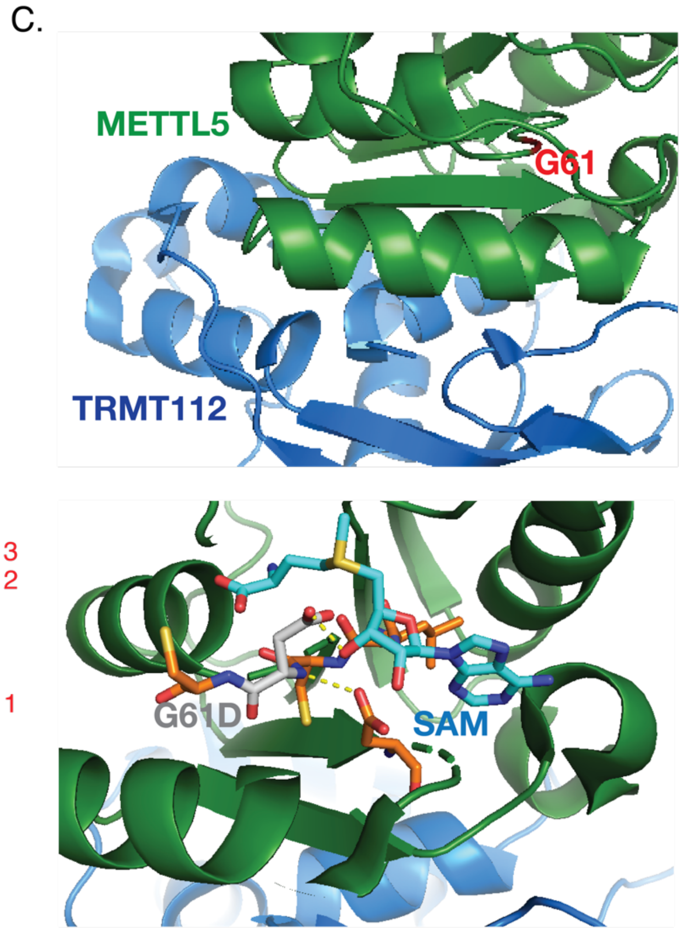

Protein	Molecular weight	Spectral count
METTL5	24 kDa	240
HMGB1	25 kDa	33
HMGB2	24 kDa	26
YWHAQ	28 kDa	21
EIF4H	27 kDa	19
YWHAZ	28 kDa	19
YWHAG	28 kDa	18
YWHAE	29 kDa	17
CACYBP	26 kDa	16
▶ TRMT112	14 kDa	15
GTF2F2	28 kDa	15
IGKV2D-29	13 kDa	12
SLC25A5	33 kDa	12
SLC25A3	40 kDa	12
FKBP3	25 kDa	11
SLC25A6	33 kDa	11

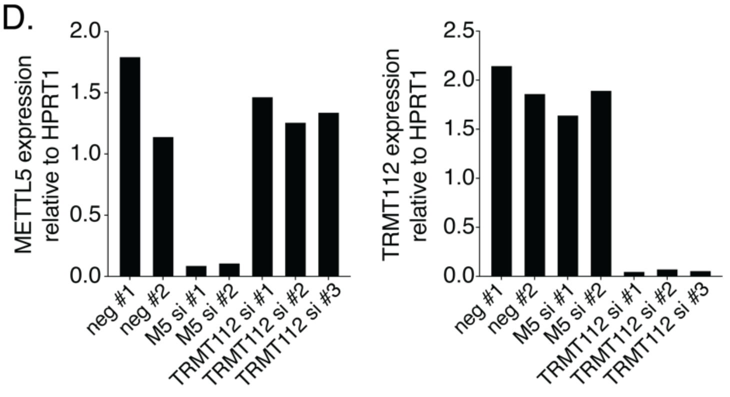

Supplementary Figure 1. Identification and investigation of METTL5-TRMT112 interactions. (A) Top: schematic of human METTL5 locus showing target locations of guide RNAs used to make METTL5 knockout HeLa and HepG2 cells in this study. Guides 2 and 3 were used to generate HeLa clone 1, while guides 2 and 32 were used to generate all other HeLa and HepG2 clones in this study. Bottom: METTL5 expression in cell lines as evaluated by western blot. Arrowhead signifies METTL5 band, * signifies background band. **(B)** Top: Silver-stained polyacrylamide protein gel with protein size marker, immunoprecipitate from pulldown with endogenous METTL5 antibody, and immunoprecipitate from pulldown with anti-FLAG antibody in Freestyle 293-F cells overexpressing FLAG-tagged METTL5. Boxed bands were cut out for proteomics analysis. Bottom: Table of top hits

from proteomics analysis of band 1 with molecular weight and spectral count of each. Suspected common contaminant proteins were removed from the list, but complete information, as well as analysis of bands 2 and 3, is available in Supplementary table 1. **(C)** METTL5-G61D human variant mutation site displayed on the METTL5-TRMT112 structure from van Tran *et al*. (PDB: 6H2U (1)). Top: Position of G61 highlighted in an unstructured loop (red) in the context of the complex with METTL5 (green) and TRMT112 (blue). Bottom: Interactions between the mutated G>D residue (gray) and S-adenosylmethionine (cyan). Neighboring residues are highlighted in orange. Images created with PyMOL v2.4.0 (2). **(D)** *METTL5* (top) and *TRMT112* (bottom) expression relative to *HPRT1* (housekeeping gene) in total RNA purified from HeLa cells treated with negative control siRNAs or siRNAs targeting *METTL5* or *TRMT112*, as evaluated by quantitative PCR. All indicated band sizes in are in kilodaltons (kDa).

Supplementary Figure 2

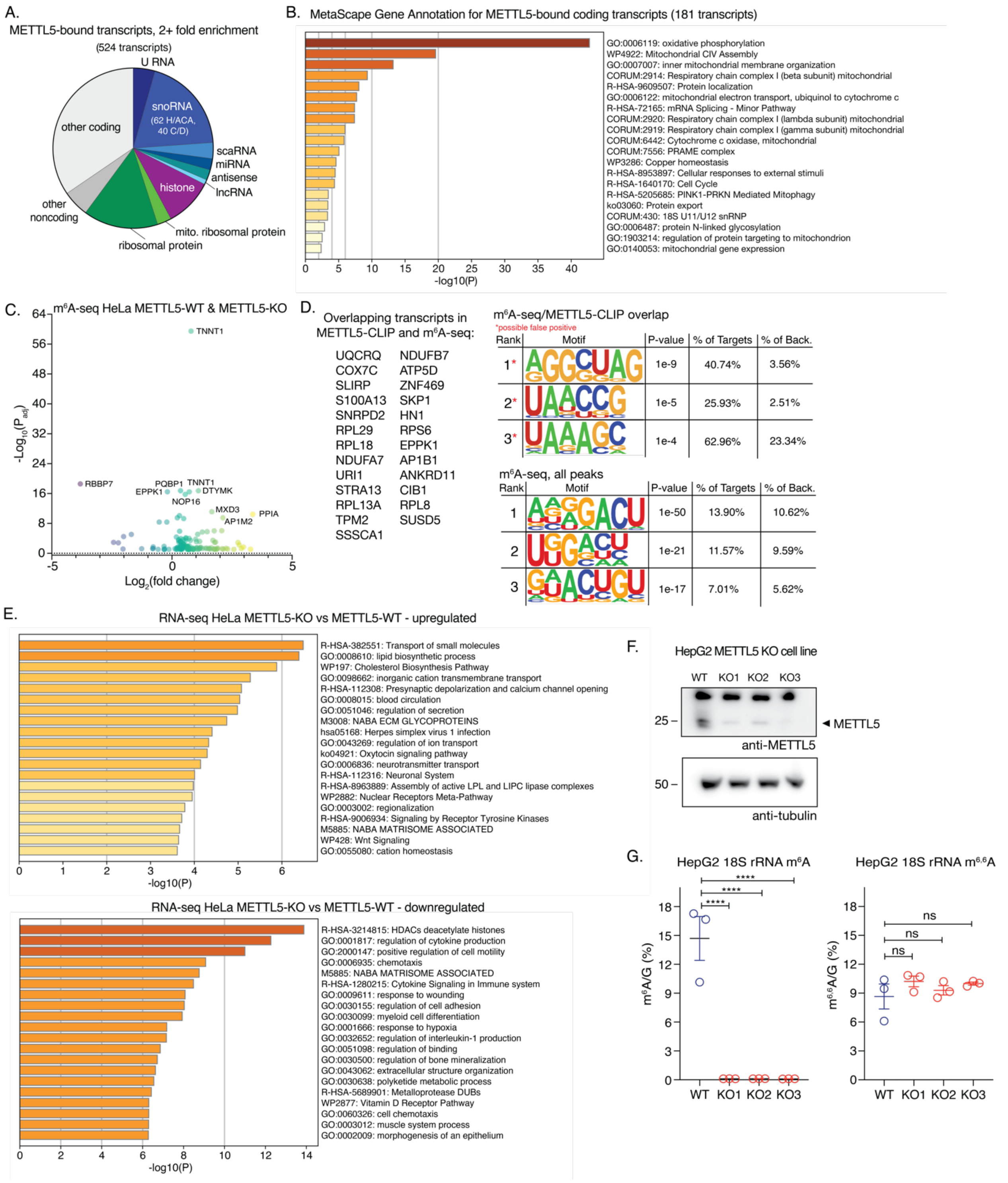

Supplementary Figure 2. Investigation of METTL5 substrates in human cells. (A) Pie chart of METTL5-bound transcripts with ≥2-fold enrichment in METTL5 immunoprecipitate after crosslinking compared to input. **(B)** MetaScape gene annotations for METTL5-bound coding transcripts. **(C)** Volcano plot of differentially methylated m^{6}A peaks between HeLa METTL5-KO and METTL5-WT cells, shown comparing negative $\log_{10}$ of the adjusted P-value with $\log_2$ fold change. **(D)** Left: List of transcripts with both differentially methylated m^{6}A peaks from MeRIP-seq and ≥2-fold enrichment in

METTL5 cross-linking and immunoprecipitation sequencing in HeLa cells. Right: Common motifs in this list of overlapping targets (top) and in all m^6A peaks from Me-RIP-seq (bottom), as predicted by HOMER (3). Back.: background (see Materials and Methods) **(E)** MetaScape gene annotation terms from transcriptionally upregulated (top) and downregulated (bottom) transcripts in RNA-seq of METTL5-KO versus METTL5-WT HeLa cells. **(F)** Western blot analysis of METTL5 levels in METTL5-WT and METTL5-KO HepG2 cells, each expanded from a single isolated clone (top) with anti-tubulin loading control (bottom). **(G)** Levels of m^6A (left) and $m^{6,6}A$ (right), normalized to G, obtained by LC-MS/MS of 40-nt probe-purified segments of 18S rRNA surrounding the m^6A1832 site from METTL5-WT and METTL5-KO HepG2 cells. Analyzed by one-way ANOVA, comparing all samples to WT, with Dunnett's test for multiple comparisons. ns: not significant, * $p<0.05$, ** $p<0.01$, *** $p<0.005$, **** $p<0.0001$. Panels B and E modified from MetaScape output (4). All indicated band sizes in western blots are in kilodaltons (kDa).

Supplementary Figure 3

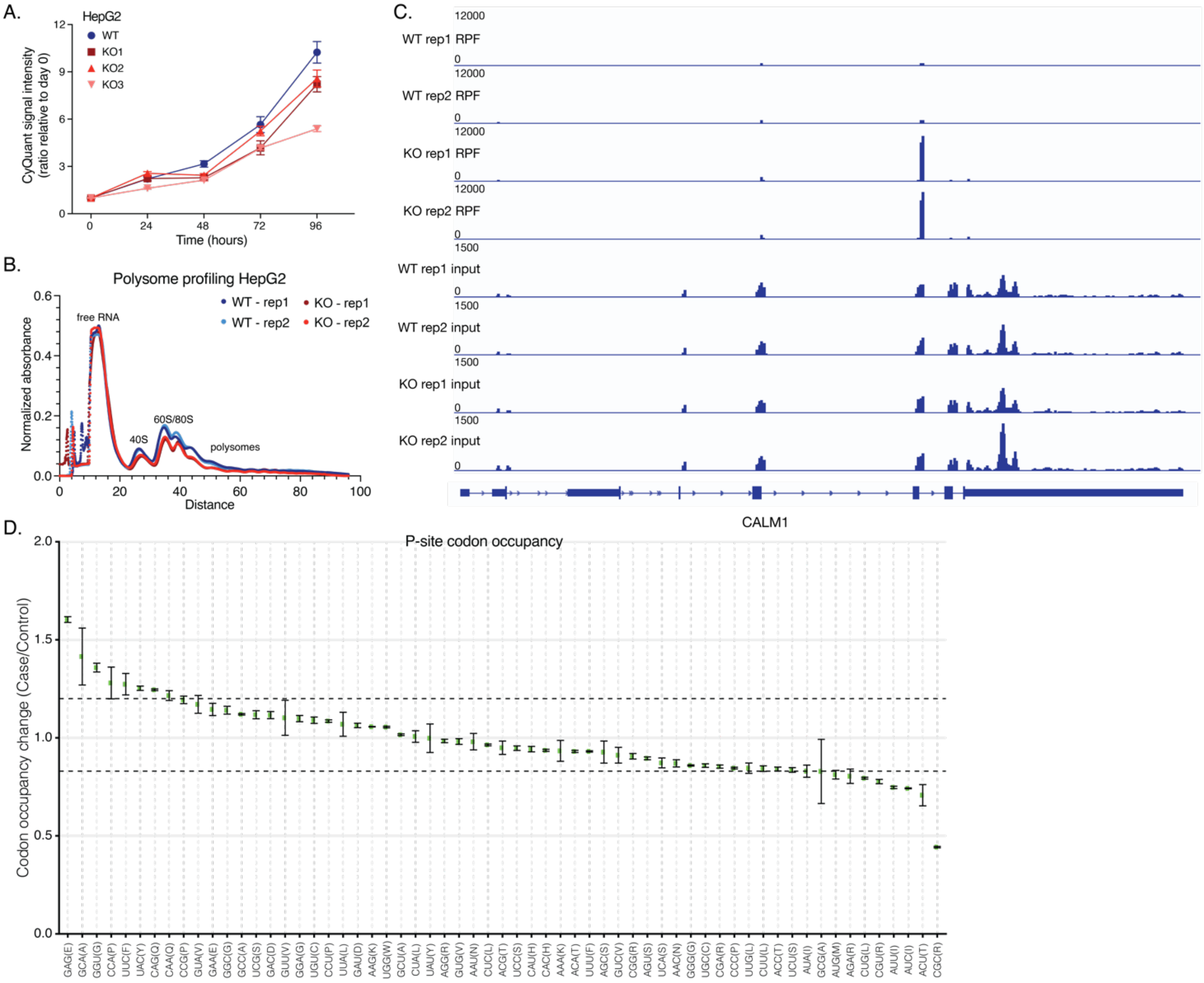

Supplementary Figure 3. The effects of METTL5 knockout on translation and cell state. (A) Cell proliferation of HepG2 METTL5-WT and METTL5-KO cell lines over time as measured by CyQuant assay. Means (squares) and SDs (bars) are indicated for 4 replicate wells per condition. **(B)** Polysome profiles from HepG2 METTL5-WT and METTL5-KO cells as measured by normalized absorbance over a 5-50% sucrose gradient. **(C)** Visualization of reads at the *CALM1* locus from input and ribosome-protected fragment (RPF) samples from HepG2 METTL5-WT and METTL5-KO cells, adapted from Integrated Genomics Viewer. **(D)** Codon occupancy changes at the P site between METTL5-KO (case) and METTL5-WT (control) HepG2 cells. D is modified from output of RiboToolKit (5).

Supplementary Figure 4

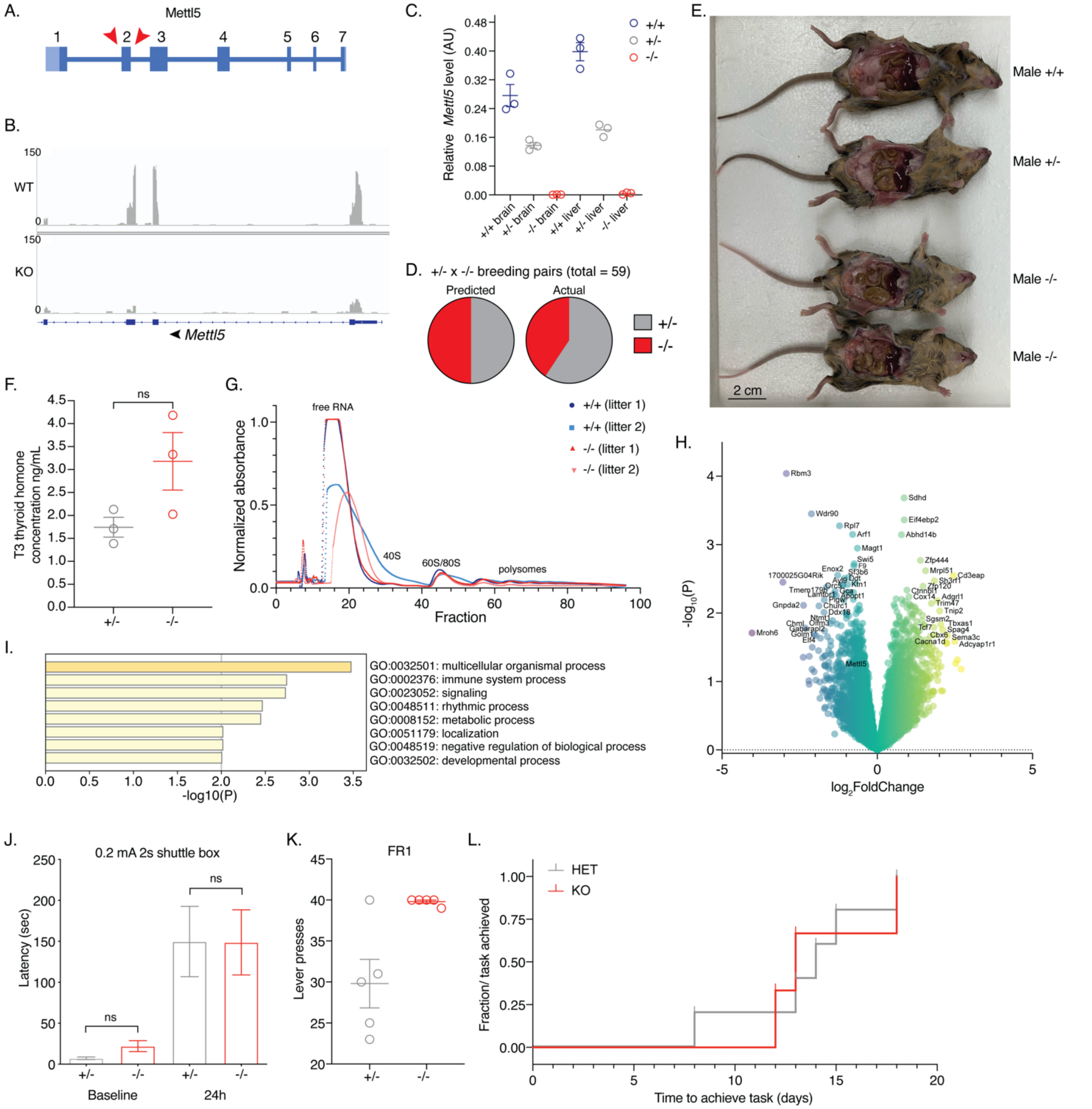

Supplementary Figure 4. Characterizations of *Mettl5*$^{-/-}$ mice. (A) Schematic of mouse *Mettl5* locus showing region targeted for removal by CRISPR-Cas9 to create KO mice for this study. **(B)** Visualization of RNA-seq reads at mouse *Mettl5* locus from a littermate pair of one *Mettl5*$^{+/-}$ and one *Mettl5*$^{-/-}$ mouse, adapted from Integrated Genomics Viewer. Exon 1 is at far right and exon 4 is on the far left; partial view shown since *Ssb* overlaps with *Mettl5*. **(C)** Relative *Mettl5* transcript level (normalized to *Hprt*) as measured by qPCR of total RNA from brains and livers of *Mettl5*$^{+/+}$, *Mettl5*$^{+/-}$ and *Mettl5*$^{-/-}$ mice. **(D)** Pie charts of the predicted (left) and actual (right) mice born of *Mettl5*$^{+/-}$, (+/-, grey) and *Mettl5*$^{-/-}$ (-/-, red) genotypes from +/- x -/- breeding pairs (total = 59 mice; 35 heterozygous, 24 knockout). **(E)** Photo of dissected male littermate mice at 8 weeks showing abdominal fat content.

(F) T3 thyroid hormone concentration (ng/mL) from enzyme-linked immunosorbent assay (ELISA) of thyroid hormone in serum of the same heterozygous and knockout mice used for RNA-seq (Figure 4E). n = 3 pairs, unpaired t-test, ns: not significant. **(G)** Polysome profiles from littermate pairs of *Mettl5*$^{+/+}$ and *Mettl5*$^{-/-}$ mouse liver tissue as measured by normalized absorbance over a 5-50% sucrose gradient. Two littermate pairs were used, indicated in the legend. **(H)** Volcano plot of the negative $\log_{10}$ of P-value versus $\log_2$(fold change) of normalized read counts from ribosome profiling of *Mettl5*$^{+/+}$ and *Mettl5*$^{-/-}$ mouse liver tissue as measured by normalized absorbance over a 5-50% sucrose gradient. **(I)** MetaScape gene annotation terms from translationally upregulated transcripts in ribosome profiling of *Mettl5*$^{+/+}$ and *Mettl5*$^{-/-}$ mouse liver tissue. **(J)** Latency in seconds for mice to move into the dark side of a shuttle box at baseline and 24 hours after training with shock to avoid that side. Data analyzed by one-way ANOVA with Sidak test for multiple comparisons, comparing *Mettl5*$^{+/-}$ to *Mettl5*$^{-/-}$ at baseline and at 24 hours; n=11 pairs, n.s.: not significant. **(K,L)** Number of lever presses on the last day (K) and time to learn task (L) by *Mettl5*$^{+/-}$ and *Mettl5*$^{-/-}$ mice in FR1 training to press a lever for food reward after food deprivation. n = 5 pairs (K, L). Log-rank (Mantel-Cox) test performed in (L) is not significant.

Supplementary Figure 5. Full images of western blots

All indicated band sizes in western blots are in kilodaltons (kDa).

Figure 1A

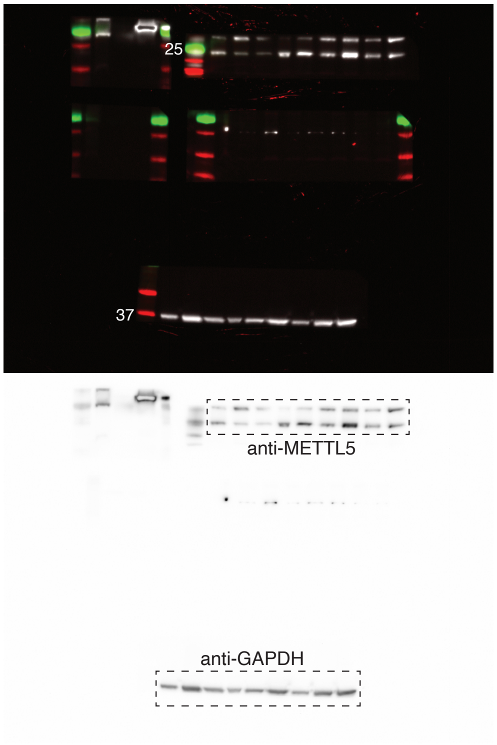

Figure 1B,D

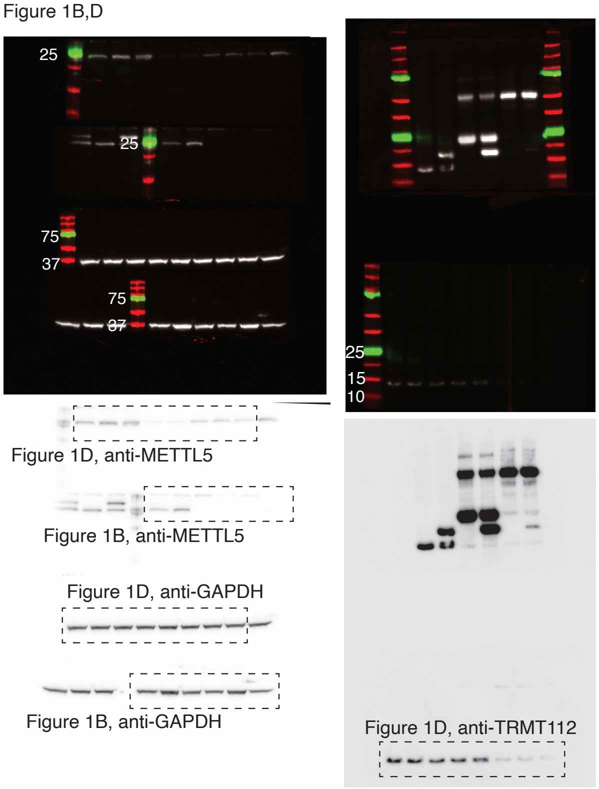

Figure 1C

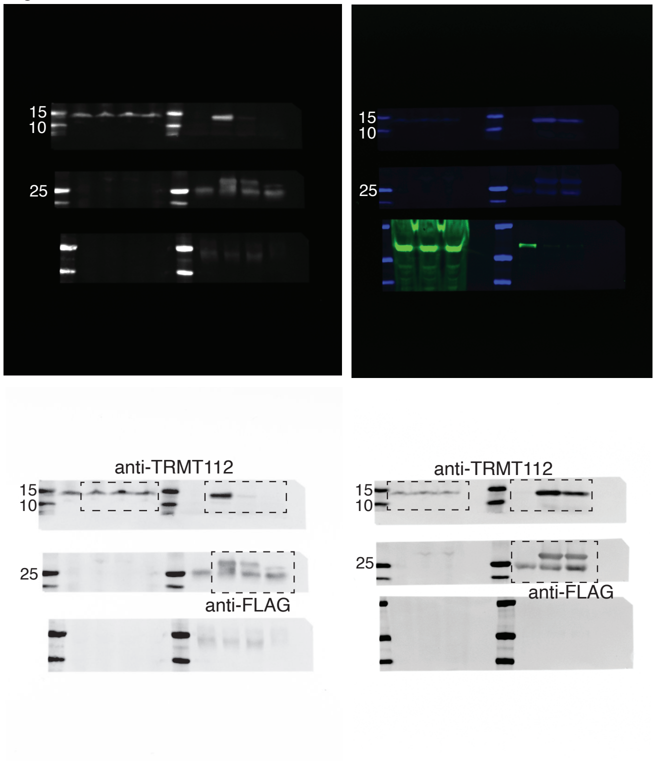

Figure 1E

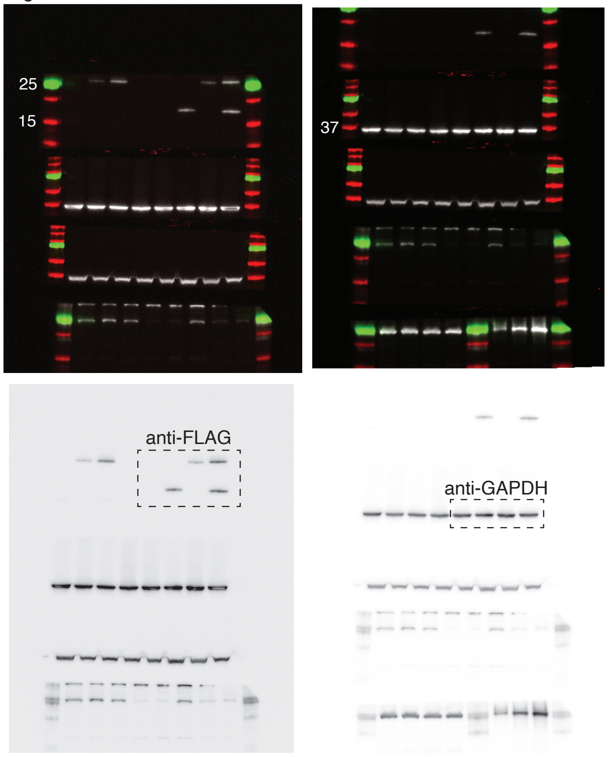

Note this is the same set of membranes, shifted vertically in the view of the camera and with different exposure times to avoid saturation of GAPDH.

Figure 2B

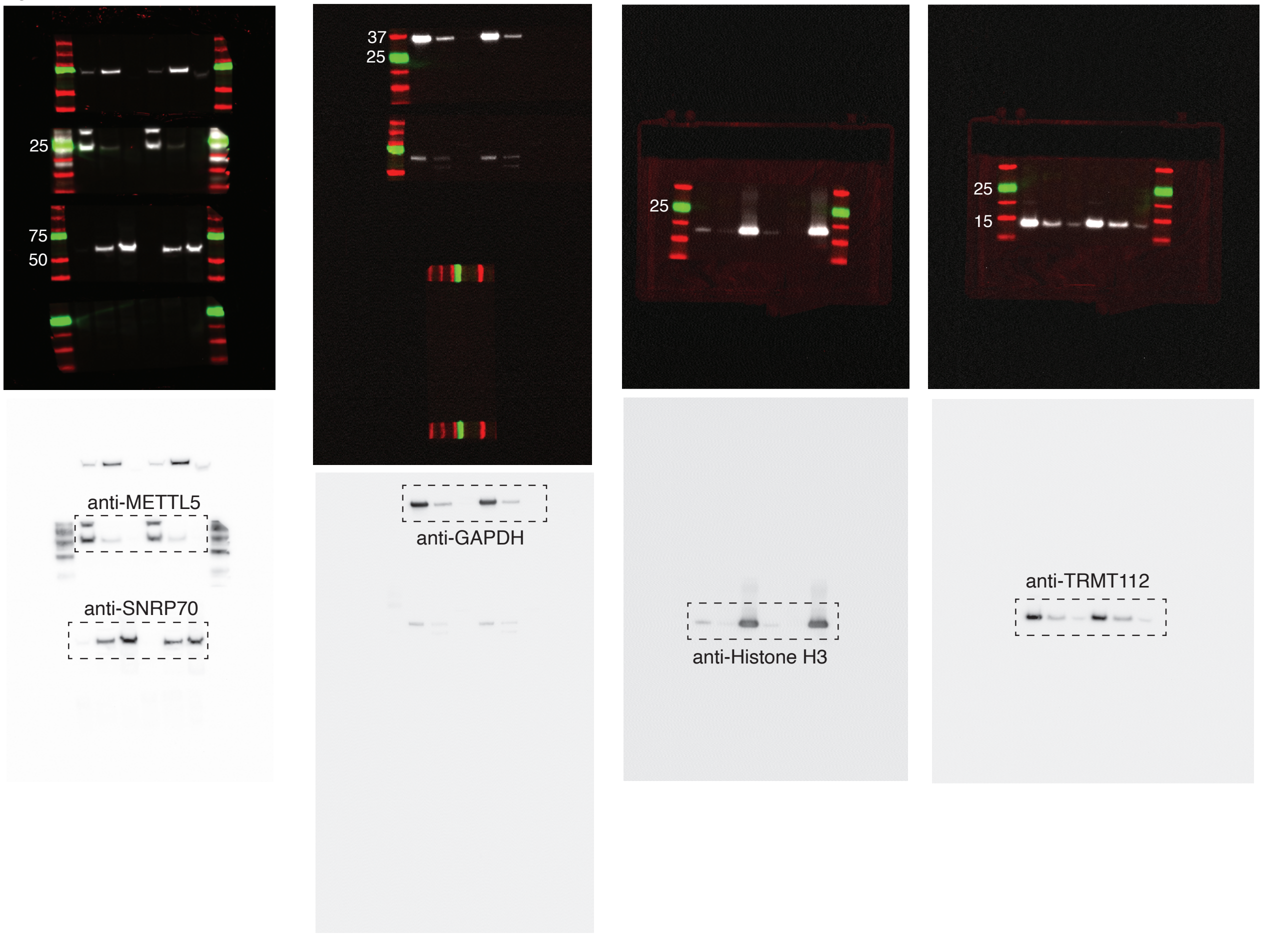
25
75
50
37
25
25
25
15
anti-METTL5
anti-SNRP70
anti-GAPDH
anti-Histone H3
anti-TRMT112

Figure 3C
METTL5 WT

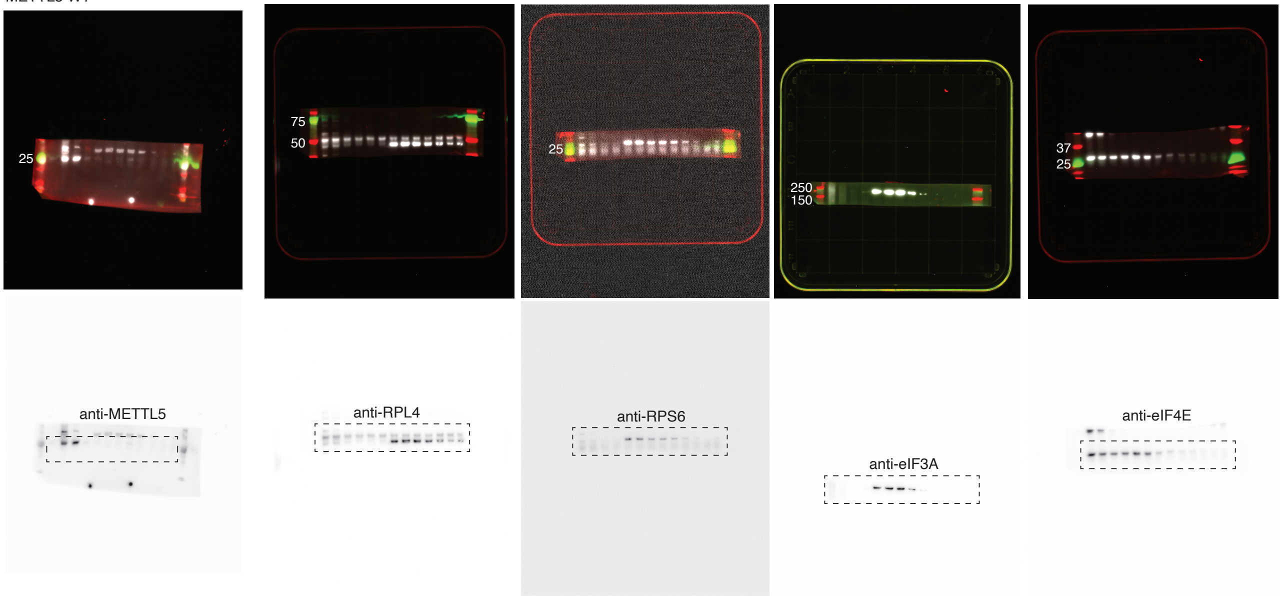

METTL5 KO

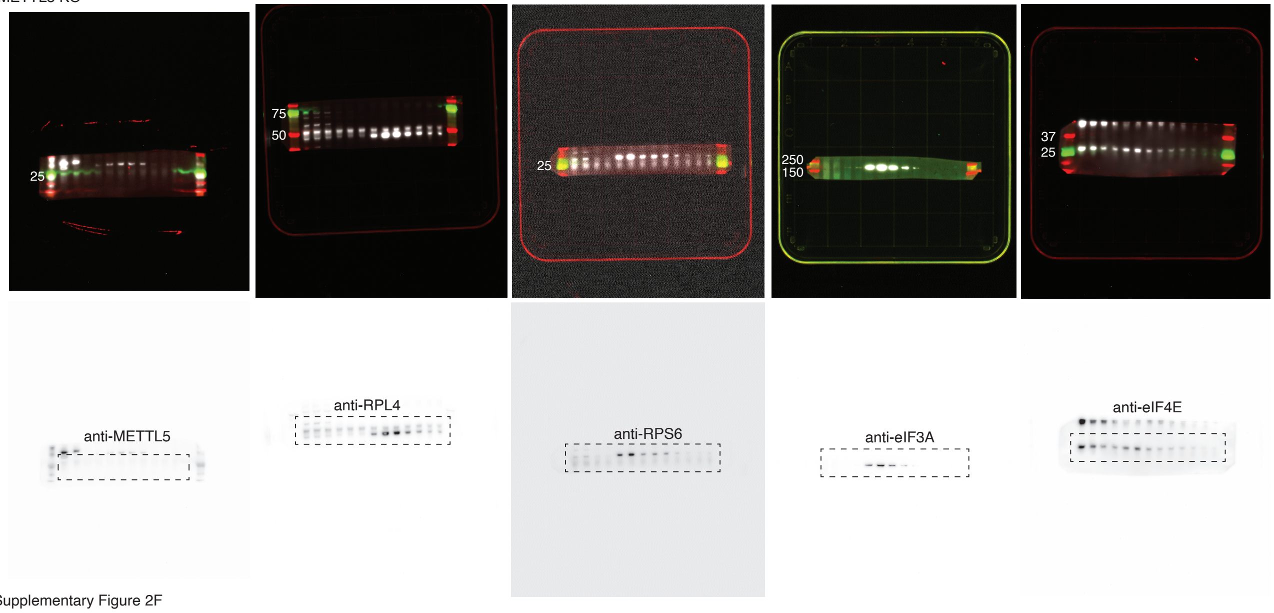

Supplementary Figure 2F

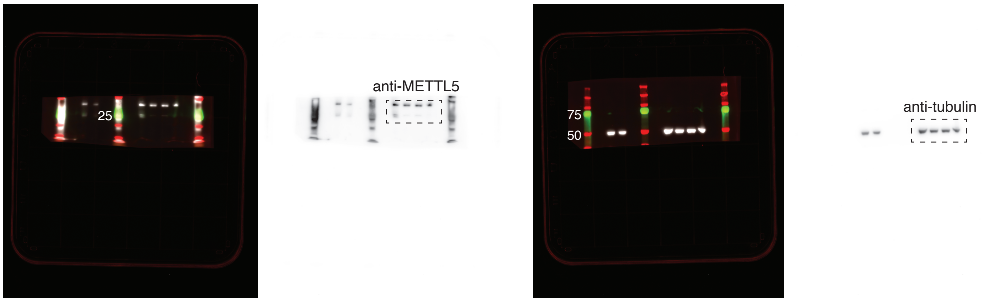

Supplementary note: Biochemical identification of METTL5

We encountered METTL5 in the course of a biochemical fractionation experiment, whose purpose was actually to identify m^1A methyltransferases. In brief, nuclear extract was fractionated over a cation exchange column, and the fractions were collected and tested for m^1A methyltransferase activity. Active fractions were then pooled and fractionated over an anion exchange column, and fractions were again collected and tested for m^1A methyltransferase activity.

Thirty 15cm dishes of HeLa cells were grown to ~80-90% confluency. Cells were washed with ice cold 1x PBS prior to harvesting cells with cell lifters. The cells were pelleted by centrifugation at 500g for 10 minutes at 4ºC (cell pellet was ~4mLs). The cell pellet was gently washed in 12mLs Buffer A and pelleted by centrifugation at 500g for 5 minutes at 4ºC. The cell pellet was gently resuspended in 8mLs Buffer A (10mM HEPES pH 7.5, 1.5mM MgCl2, 10mM KCl, 1x SigmaFast Protease Inhibitor cocktail [Sigma]) and incubated on ice for 10 minutes. The cell suspension was then lysed with a dounce with a loose pestle (20 times, while on ice). Nuclei were pelleted by centrifugation at 500g for 5 minutes at 4ºC, and the supernatant (cytoplasm) was removed. The nuclear pellet was then resuspended and lysed in 1.5mLs of Buffer C (20mM HEPES pH 7.5, 25% glycerol, 0.42M NaCl, 1.5mM MgCl2, 0.2mM EDTA, 0.5mM DTT, 1x SigmaFast Protease Inhibitor cocktail [Sigma]), rotating for 30 minutes at 4ºC. Nuclear lysate was spun at 20,000g for 30 minutes at 4ºC to pellet debris, and the supernatant (~2mLs) was dialyzed into 20mM HEPES pH 7.5, 50mM NaCl, 1.5mM $MgCl_2$, 0.5mM EDTA using a 3500 Da molecular weight cut off cassette for 6 hours at 4ºC.

The dialyzed lysate was then fractionated over a Fast Flow SP (GE Healthcare) column in 20mM HEPES pH 7.5,1.5mM $MgCl_2$, 0.5mM EDTA using a gradient from 50mM to 1M NaCl. Fractions were collected and concentrated and exchanged into methyltransferase buffer (MT buffer: 50mM Tris pH 8.0, 10% glycerol, 1.5mM $MgCl_2$, 0.5mM EDTA, and 100mM NH_4SO_4) through 10,000 Da molecular weight cut off spin filters. Each fraction was tested for methyltransferase activity by combining 20uL fraction with 1mM d_3SAM, 3% v/v SUPERasin RNase inhibitor, and 750ng of an RNA probe (5'-CUGACCCUCGCCUGCACCCGCCCCGAGAAGCCGAG-biotin-3'). Reactions were incubated at 30ºC for 30 minutes and then snap frozen and stored at -80ºC until clean-up and processing for LC-MS/MS analysis.

For clean-up, 1mM EDTA and 1uL Proteinase K (Sigma, #P4850) was added to each 30uL methyltransferase reaction, and incubated for 1 hour at 37ºC. The samples were then diluted into 500uL IP buffer (5mM Tris pH 7.4, 1M NaCl, 1mM EDTA, 1% SUPERasin). 10uL MyOne C1 streptavidin beads per sample were washed into IP buffer and added to each sample. Samples with beads were rotated for 2 hours at 4ºC, after which beads were washed twice with IP buffer and eluted with Trizol (twice for 5 minutes at room temperature, shaking at 1000rpm). The eluates were then cleaned up with the DirectZol Micro clean up kit (Zymo). The samples were then digested into nucleosides and analyzed by LC-MS/MS as described in the 'LC-MS/MS' section (see Materials and Methods).

Active fractions were then subjected to a second fractionation procedure, this time using a Fast Flow Q column (GE Healthcare) in 20mM Tris pH 7.5,1.5mM $MgCl_2$, 0.5mM EDTA using a gradient from 50mM to 1M NaCl (Supp. Fig. 6A). Methyltransferase reactions were set up with each fraction as described above (Supp. Fig. 6B) . After analysis of this second round of fractions for d_3m^1A activity by LC-MS/MS, fractions were selected for analysis by TMT11-LC-MS/MS proteomics analysis (see Materials and Methods: TMT11-LC-MS/MS; Table S8), which yielded quantitation of numerous proteins in each active fraction. To narrow down possible candidates, the abundance of

all putative RNA modification enzymes was normalized by the total protein concentration of the original protein fractions, and plotted in Supp. Fig. 6C. METTL5 appeared as an overrepresented protein in active fraction F11, and was pursued further as described in this manuscript.

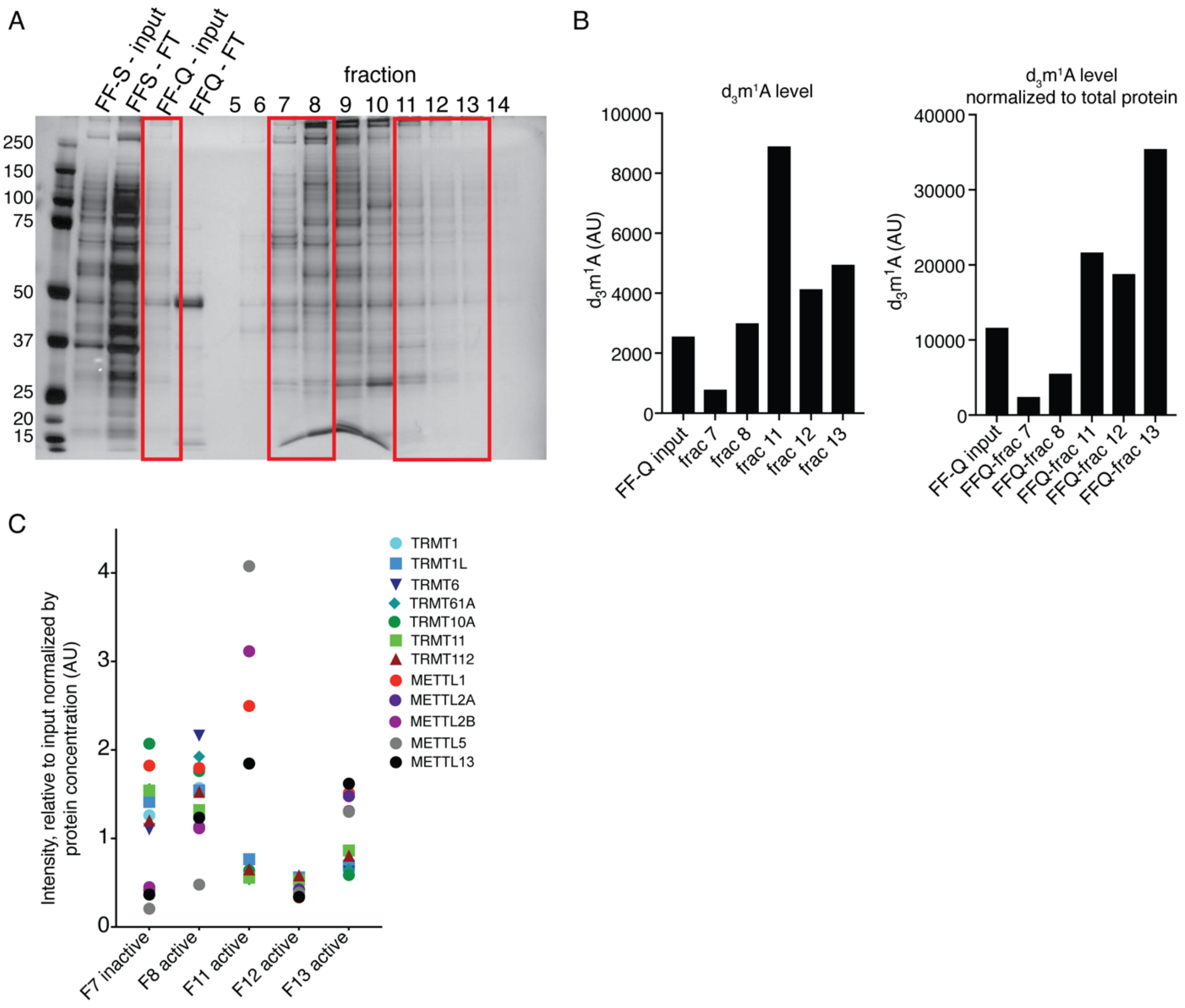

Supplementary Figure 6. Biochemical fractionation to identify METTL5. (A) SDS-PAGE analysis of selected fractions, visualized by silver stain. The lanes indicated in red boxes correspond to those whose activity is shown in **(B)** and which were analyzed by TMT11-LC-MS/MS in **(C)**. FF-S, Fast Flow SP column; FF-Q, Fast Flow Q column.

REFERENCES

1. van Tran, N., Ernst, F. G. M., Hawley, B. R., Zorbas, C., Ulryck, N., Hackert, P., Bohnsack, K. E., Bohnsack, M. T., Jaffrey, S. R., Graille, M., and Lafontaine, D. L. J. (2019) The human 18S rRNA m6A methyltransferase METTL5 is stabilized by TRMT112. *Nucleic Acids Res* **47**, 7719-7733
2. Schrodinger LLC, D. W. (2020) The PyMOL Molecular Graphics System, Version 2.0.
3. Heinz, S., Benner, C., Spann, N., Bertolino, E., Lin, Y. C., Laslo, P., Cheng, J. X., Murre, C., Singh, H., and Glass, C. K. (2010) Simple combinations of lineage-determining transcription factors prime cis-regulatory elements required for macrophage and B cell identities. *Mol Cell* **38**, 576-589
4. Zhou, Y., Zhou, B., Pache, L., Chang, M., Khodabakhshi, A. H., Tanaseichuk, O., Benner, C., and Chanda, S. K. (2019) Metascape provides a biologist-oriented resource for the analysis of systems-level datasets. *Nat Commun* **10**, 1523
5. Liu, Q., Shvarts, T., Sliz, P., and Gregory, R. I. (2020) RiboToolkit: an integrated platform for analysis and annotation of ribosome profiling data to decode mRNA translation at codon resolution. *Nucleic Acids Res* **48**, W218-W229
